# Methyltransferase like 7B is a potential therapeutic target for reversing EGFR-TKIs resistance in lung adenocarcinoma

**DOI:** 10.1186/s12943-022-01519-7

**Published:** 2022-02-10

**Authors:** Huibin Song, Dongcheng Liu, Lingwei Wang, Kaisheng Liu, Chen Chen, Le Wang, Yi Ren, Bing Ju, Fuhua Zhong, Xingyu Jiang, Guangsuo Wang, Zhe-Sheng Chen, Chang Zou

**Affiliations:** 1 Department of Clinical Medical Research Center, The First Affiliated Hospital of Southern, The 2nd Clinical Medical College (Shenzhen People’s Hospital) of Jinan University, University of Science and Technology, Shenzhen, 518020 China; 2 Integrated Chinese and Western Medicine Postdoctoral Research Station, Jinan University, Guangzhou, 510632 China; 3 Department of Respiratory and Critical Care Medicine, First Affiliated Hospital of Southern, University of Science and Technology, Second Clinical Medical College of Jinan University, Shenzhen People’s Hospital, Shenzhen Institute of Respiratory Diseases, Shenzhen, 518020 China; 4 Department of Biomedical Engineering, Southern University of Science and Technology, Nanshan District, No. 1088 Xueyuan Rd, Shenzhen, Guangdong China; 5 Department of Thoracic Surgery, The First Affiliated Hospital of Southern, University of Sciences and Technology, Shenzhen People’s Hospital, Shenzhen, China; 6 College of Pharmacy and Health Sciences, St. John’s University, New York, USA; 7 Shenzhen Public Service Platform On Tumor Precision Medicine and Molecular Diagnosis, the Second Clinical Medical College of Jinan University, Shenzhen People’s Hospital, Shenzhen, 518020 China; 8 School of Life and Health Sciences, The Chinese University of Hong Kong, Shenzhen, China

**Keywords:** METTL7B, TKIs resistance, Glutathione metabolism, m^6^A modification, Lung adenocarcinomas

## Abstract

**Background:**

Identification of potential novel targets for reversing resistance to Epidermal Growth Factor Receptor (EGFR)-tyrosine kinase inhibitors (EGFR-TKIs) holds great promise for the treatment of relapsed lung adenocarcinoma (LUAD). In the present study, we aim to investigate the role of methyltransferase-like 7B (METTL7B) in inducing EGFR-TKIs resistance in LUAD and whether it could be a therapeutic target for reversing the resistance.

**Methods:**

METTL7B-overexpressed LUAD cell lines, gefitinib and osimertinib-resistant Cell-Derived tumor Xenograft (CDX) and Patient-Derived tumor Xenograft (PDX) mouse models were employed to evaluate the role of METTL7B in TKIs resistance. Ultraperformance liquid chromatography-tandem mass spectrometer (UPLC-MS/MS) was used to identify the metabolites regulated by METTL7B. Methylated RNA immunoprecipitation (MeRIP)-qPCR analysis was performed to measure the N^6^-methyladenosine (m^6^A) status of mRNA of METTL7B targeted genes. Gold nanocluster-assisted delivery of siRNA targeting METTL7B (GNC-siMETTL7B) was applied to evaluate the effect of METTL7B in TKIs resistance.

**Results:**

Increased expression of METTL7B was found in TKIs-resistant LUAD cells and overexpression of METTL7B in LUAD cells induced TKIs resistance both in vitro and in vivo. Activated ROS-metabolism was identified in METTL7B-overexpressed LUAD cells, accompanied with upregulated protein level of GPX4, HMOX1 and SOD1 and their enzymatic activities. Globally elevated m^6^A levels were found in METTL7B-overexpressed LUAD cells, which was reduced by knock-down of METTL7B. METTL7B induced m^6^A modification of GPX4, HMOX1 and SOD1 mRNA. Knock-down of METTL7B by siRNA re-sensitized LUAD cells to gefitinib and osimertinib both in vitro and in vivo.

**Conclusions:**

This study uncovered a new critical link in METTL7B, glutathione metabolism and drug resistance. Our findings demonstrated that METTL7B inhibitors could be used for reversing TKIs resistance in LUAD patients.

**Supplementary Information:**

The online version contains supplementary material available at 10.1186/s12943-022-01519-7.

## Background

Lung adenocarcinoma (LUAD) is one of the most prevalent malignant tumors with high morbidity and mortality worldwide [1]. Precision medicine based on targeting epidermal growth factor receptor (EGFR) has contributed greatly to the improvement of LUAD treatment in the past decade [2]. The EGFR tyrosine kinase inhibitors (TKIs) have been widely used in clinical practice and significantly prolonged the survival of patients due to their advantages such as rapid absorption, high efficacy and good safety profiles [3]. However, up to 60% of patients ultimately develop drug resistance over a period of 9 to 13 months of using the first- or third-generation TKIs (gefitinib or osimertinib) [4]. One of the well-known mechanisms of developing drug resistance is the genetic mutation of EGFR (e.g. T790M or C797S), in which the mutated protein (either from acquired under selective stress or pre-existed) escapes from the interaction and inhibition of TKIs, resulting in cancer progression and drug resistance [5]. A number of studies have shown that EGFR-independent signaling pathways, including amplification of various receptor tyrosine kinases, activation of bypass signals and epithelial-mesenchymal transition (EMT), also play significant roles in TKIs resistance [6, 7]. It is reported that about 40%-50% EGFR-TKIs resistance is related to EGFR-independent signaling pathway [8–10]. Therefore, elucidation of new targets independent of EGFR and in-depth understanding of TKIs resistance in LUAD holds not only basic research value but also great clinical significance.

Reactive oxygen species (ROS), including radicals and ions, are highly reactive molecules. ROS are excessively increased under pathophysiological conditions such as hypoxia, chemical stress, drug treatment, and might cause oxidative damage to DNA and genomic instability [11]. Accumulating evidence indicate that intra-tumoral redox homeostasis is involved in tumor development and drug resistance [12, 13]. Previous studies had demonstrated the strong correlations between ROS and EGFR-TKIs resistance in lung cancer [14, 15]. Therefore, blocking oxidative stress-mediated signaling pathways by reducing antioxidant enzymes is a reasonable strategy for reversing TKIs drug resistance [16].

Methyltransferase-like 7B (METTL7B) is a member of the methyltransferase-like family which contains a methyltransferase domain [17]. Studies from our group and others had identified that METTL7B was involved in tumor occurrence, development, invasion, and migration in various malignant tumors [18–20]. However, the role of METTL7B in tumor drug resistance and the underlying mechanism remains unknown. Here, we found that the expression of METTL7B was significantly higher in TKIs-resistant cells in comparison to TKIs-sensitive cells. Moreover, overexpression of METTL7B induced resistance to gefitinib and osimertinib in LUAD cells both in vitro and in vivo. METTL7B enhanced the expressions of antioxidant enzymes (including SOD1, GPX4 and HMOX1) as well as their enzymatic activities in the ROS-scavenging signaling pathway. Interestingly, MeRIP-qPCR analysis showed that instead of regulating the classic upstream transcription factor NRF2, METTL7B directly upregulated the expression of these antioxidant enzymes through mRNA m^6^A modification. Furthermore, knock-down of METTL7B by GNC-siRNA re-sensitized LUAD cells to gefitinib and osimertinib both in vitro and in vivo. Overall, this study provides new insights into the molecular targeted therapy and potential target for reversing EGFR-TKIs resistance in LUAD.

## Methods

### Patients and tumor specimens

The LUAD tissues used for PDX model was obtained from the Second Clinical Medical College of Jinan University & Shenzhen People’s Hospital (Shenzhen, China). The patient was clinically and pathologically diagnosed at the Department of thoracic surgery, the Second Clinical Medical College of Jinan University & Shenzhen People’s Hospital. This study was approved by the Ethics Committee of Shenzhen People’s Hospital. Written informed consents were obtained from the participants.

### Cell lines and culture conditions

TKI-sensitive LUAD cell lines PC9 and HCC827, gefitinib-resistant LUAD cell lines PC9-GR and H1975, osimertinib-resistant LUAD cell lines PC9-OR were kindly provided by Dr. Wenfeng Fang at the Sun Yat-sen University Cancer Center, Guangzhou, China. Cells were cultured in RPMI 1640 medium (Invitrogen, USA) containing 10% FBS and 1% penicillin–streptomycin at 37 ℃ with 5% CO_2_. PC9 and HCC827 cells harbored EGFR exon 19 deletion. H1975 cells harbored EGFR L858R and T790M mutation.

### Lentivirus production and transduction

For lentiviruses that mediated shRNA knock-down, METTL7B overexpression and dox-inducible METTL7B expression system, pLKO.1-GFP, pLV-METTL7B-Flag or pInducer-METTL7B construct together with packing plasmids psPAX2 and pMD2.G were co-transfected into 293 T cells. Viruses were collected at 48 h and 72 h after transfection. For shRNA knock-down of METTL7B, viruses were added to PC9-GR, H1975 or PC9-OR cells with polybrene (8 μg/mL, Sigma). 48 h after infection, puromycin was added to the culture medium for stable cell selection and doxycycline (1 μm, Selleck Chemicals) was added to the culture medium for METTL7B overexpression for 72 h. The shRNA sequences targeting human METTL7B were as follow, shMETTL7B-1: 5’- CAGGGCAATCTCTAACTTCA-3’, shMETTL7B-2: 5’- GGGCAATCTCTAACTTCAATC-3’.

### Reagents and cell viability assay

Gefitinib and osimertinib (Selleck, USA) were dissolved in Dimethyl sulfoxide (DMSO, Sigma-Aldrich, Germany) at a stock concentration of 20 mM. N-acetyl-L-cysteine (NAC, Beyotime, China) and Glutathione (GSH, Beyotime, China) were reconstituted in ddH_2_O at a stock concentration of 1 M. Cells were plated at a density of 3,000 cells/well in 96 well plates. Drugs, siRNAs or vehicle control were added to the medium and treated for 72 h. Cell viability assays were performed by Cell Counting Kit-8 (Dojindo, Mashikimachi, Japan) according to the manufacturer’s instructions. The half maximal inhibitory concentration (IC_50_) values were generated and compared using GraphPad Prism.

### RNA extraction and Real-time quantitative PCR assays

Total RNA was extracted from cells using TRIzol Reagent (Invitrogen, USA), and cDNA was synthesized from 1 μg of RNA with the M-MLV Reverse Transcriptase Kit (Promega, USA) as recommended by the manufacturer. Real-time quantitative PCR were performed with Bio-Rad iQ5 Real Time PCR System. The primers sequences used in this study were listed in Additional file 1: Table S1. The expression level of each individual transcript was normalized to GAPDH.

### Western blot analysis

The cells were washed with PBS, lysed in radio-immunoprecipitation assay (RIPA) buffer containing 1 mM PMSF (Beyotime, China) and placed on ice for 30 min. Then, cells were centrifuged at 13,800 × g for 10 min, and the protein concentration was determined using BCA Protein Assay Kit (Thermo Fisher, USA). Proteins were separated by 10% SDS-PAGE gel and electro-transferred to polyvinylidene difluoride membrane (Millipore, USA). The membranes were blocked with 5% nonfat milk in 1 × TBST and 0.1% Tween 20 for 1 h at room temperature, and incubated at 4 ℃ overnight with the following primary antibodies: Anti-METTL7B (A7200, Abclonal Technology, China); Anti-FLAG (AE063, Abclonal Technology, China); Anti-GPX4 (ab125066, Abcam, England); Anti-SOD1 (A12537, Abclonal Technology, China); Anti-NRF2 (A0674, Abclonal Technology, China); Anti-HMOX1 (A19062, Abclonal Technology, China); Anti-SOD2 (ab68155, Abcam, England); Anti-GPX1 (ab108427, Abcam, England); Anti-GPX2 (ab140130, Abcam, England); Anti-GPX7 (A3902, Abclonal Technology, China); and Anti-GPX8 (A20390, Abclonal Technology, China). Anti-α-tubulin (#2125, CST, USA) was used as an internal loading control. After incubation with corresponding secondary antibodies (CST, USA), the membranes were incubated with ECL substrate (Thermo Fisher, USA).

### Metabolomics analysis based on UPLC-MS/MS

Cells were washed with cold PBS and collected using a cell scraper. Next, 100 mg of the sample was transferred to a 2 mL centrifuge tube containing 0.3 mL of ethanol, ultrasonicated for 30 min at 25 ℃ and centrifuged at 12,000 rpm for 10 min. The supernatant was filtered through a 0.22 µm membrane. Thirty microliters of filtrate were obtained from each supernatant and mixed to make the quality control sample. The remaining samples were tested by UPLC-MS/MS. For UPLC, chromatographic separation was accomplished in an Thermo Vanquish system equipped with an ACQUITY UPLC® HSS T3 (150 × 2.1 mm, 1.8 μm, Waters) column maintained at 40 ℃. The temperature of the autosampler was 8 ℃. Gradient elution of analytes was carried out with 0.1% formic acid in water (B2) and 0.1% formic acid in acetonitrile (A2) or 5 mM ammonium formate in water (B1) and acetonitrile (A1) at a flow rate of 0.25 mL/min. Injection of 2 μL of each sample was done after equilibration. For MS, The ESI-MSn experiments were executed on the Thermo Q Exactive Focus mass spectrometer with the spray voltage of 3.8 kV and -2.5 kV in positive and negative modes, respectively. Sheath gas and auxiliary gas were set at 30 and 10 arbitrary units, respectively. The capillary temperature was 325 ℃. The analyzer scanned over a mass range of m/z 81–1 000 for full scan at a mass resolution of 70 000. Data dependent acquisition (DDA) MS/MS experiments were performed with HCD scan. The normalized collision energy was 30 eV. Dynamic exclusion was implemented to remove some unnecessary information in the MS/MS spectra.

### Assessment of oxidative stress

For detection of ROS, intracellular ROS levels were measured using a Reactive Oxygen Species Assay Kit (Beyotime Biotechnology, China). After METTL7B was overexpressed or knocked down, the cells were incubated with DCFH-DA for 30 min at 37 °C and measured at 488 nm excitation and 525 nm emission by BioTek CYTATION 5 image reader. For detection of reactive nitrogen species (RNS), intracellular RNS levels were measured using a Reactive Nitrogen Species Assay Kit (Bestbio, China). After METTL7B was overexpressed or knocked down, the cells were incubated with BBoxiProbe O52 for 30 min at 37 °C and measured at 490 nm excitation and 516 nm emission by BioTek CYTATION 5 image reader. For detection of SOD enzymatic activity, Cu/Zn-SOD and Mn-SOD Assay Kit with WST-8 (Beyotime Institute of Biotechnology, Shanghai, China) was used. After METTL7B was overexpressed or knocked down, the proteins of cells were extracted and incubated with WST-8/enzyme working solution for 30 min at 37 °C and measured at 450 nm by BioTek CYTATION 5 image reader. For detection of GPX enzymatic activity and GSH, Glutathione Peroxidase Assay Kit with NADPH was used. After METTL7B was overexpressed or knocked down, the proteins of cells were extracted and incubated with GPX detection working solution for 15 min at 25 °C, then measured at 340 nm continuously for 5 min by BioTek CYTATION 5 image reader after peroxide was added.

### Animal study approval and in vivo xenograft assay

Animal study was approved by the Jinan University Institutional Animal Care and Use Committee. Experimental procedures were performed in accordance with the Guide for the Care and Use of Laboratory Animals (National Institutes of Health Publication No. 80–23) and according to the institutional ethical guidelines for animal experiments. Female BALB/c nu/nu mice and NCG mice (4–5 weeks old) were purchased from Gem Pharmatech (Nanjing, China). For PDX mouse model, tumor tissue harboring EGFR L858R or exon19 del mutation from LUAD patient were subcutaneously inoculated into NCG mice (Additional file 4: Figure S1). The tumor tissue was cut into scraps and passaged two generations to stabilize its inheritable information. After the tumor of NCG mice were stably formed, the mice were divided into two groups: one group for control and the other group was treated with TKIs (gefitinib and osimertinib) continuously until the tumors were resistant to TKIs. For Cell-Derived tumor Xenograft (CDX) mouse models, PC9, PC9-GR and PC9-OR cells (1 × 10^7^ cells/mice) were subcutaneously inoculated into BALB/c nu/nu mice. After the tumors were stably formed, mice were treated with TKIs (gefitinib and osimertinib) and the tumor volumes (V = L × W^2^ /2) was measured at a two-day interval. At the beginning of treatment with TKIs, tumor sizes in both CDX and PDX models were decreased significantly. After continuous treatment with TKIs, the volume of tumors started to regain, indicating that the xenograft mouse model that resistant to TKIs had been successfully established.

### Immunohistochemistry

The tissue sections were cultured overnight with Anti-METTL7B (A7200, Abclonal Technology, China); Anti-GPX4 (ab125066, Abcam, England); Anti-SOD1 (A12537, Abclonal Technology, China); Anti-HMOX1 (A19062, Abclonal Technology, China) at 4 °C and then cultured with secondary antibody and horseradish peroxidase. All immunohistochemistry (IHC) samples were evaluated by two independent pathologists who were unaware of the source of samples and the results of the subjects. The immunohistochemical images were captured using the BioTek CYTATION 5 image reader. Each core was given a score from 0 to 3 + depending on the METTL7B, GPX4, SOD1 or HMOX1 expression in tumor cells. 0 was given for expression in less than 5% of the tumor cells, 1 + for expression in 5–50% of the tumor cells, 2 + for expression in 50–75%, 3 + for expression in more than 75% of tumor cells. For each tumor, the final score was based on the mean scores of all tumor cores. The statistical evaluation was based on the product of dyeing rate and dyeing intensity.

### m^6^A MS

Polyadenylated mRNA was purified by GenElute™ mRNA Miniprep Kit (Sigma, St. Louis, MO) from the previously isolated total RNA. Totally, 300 ng of the purified mRNA were digested by nuclease P1 (2U, Wako) in 25 μL of buffer containing 20 mM NH_4_OAC (pH 5.3) at 42 °C for 2 h. NH_4_HCO_3_ (1 M, 3 μL, freshly made) and alkaline phosphatase (1U, Sigma) were added and incubated at 37 °C for 2 h. The samples were then diluted to 50 μL with water and filtered (0.22 μm pore size, Millipore) for the following LC–MS analysis. 2 μL of the digested RNA solution was injected into UltiMate 3000 HPLC system (Thermo Scientific) coupled with QTRAP 6500 mass spectrometry (Sciex) for MRM (multiple reaction monitoring) analysis. Nucleosides were separated by AQ-C18 column (2.1 × 250 mm i.d., 5 μm, Welch) with a flow rate of 0.15 mL/min, the gradient of 1 min 100% A (water with 0.1% formic acid), 1–10 min 0%-90% B (acetonitrile with 0.1% formic acid), 2 min 90% B, and 8 min 100% A was used. The column temperature was kept at 25 °C. Nucleosides were quantified by the nucleosides-to-base ion mass transitions of m/z 268 to 136 (A) and m/z 282 to 150 (m^6^A). The m^6^A/A ratio in mRNA was calculated by the standard curve obtained from pure nucleoside standards.

### m^6^A dot blot

The m^6^A dot blot assay was performed as previously described [21]. The total RNA samples were loaded to Hybond-N + membrane (GE Healthcare, UK) and UV crossed with the nylon membrane. The membrane was then blocked with 5% nonfat milk for 1 h and incubated with m^6^A antibody (A19841, Ablconal Technology, China) at 4℃, overnight. After incubating with horseradish peroxidase-conjugated anti-mouse IgG, the membrane was visualized with the ECL detection system. The same amount of total RNA samples was spotted on the membrane and stained with 0.02% methylene blue (MB) in 0.3 M sodium acetate (pH = 5.2). The results of m^6^A level were shown in the form of relative density normalized to methylene blue staining density of the m^6^A dot blot.

### Methylated RNA immunoprecipitation-PCR (MeRIP-qPCR)

Total RNA was extracted using Trizol reagent, and mRNA was purified using GenEluteTM mRNA Miniprep Kit (Sigma, Louis, MO). RNA fragmentation reagents (NEB, Hertfordshire, UK) were used to randomly fragment RNA. The specific anti-m^6^A antibody (NEB, Hertfordshire, UK) was applied for m^6^A immunoprecipitation. Anti-m^6^A antibody was pre-bound to Protein G magnetic beads in reaction buffer for 30 min. The fragmented mRNA was incubated with m^6^A-antibody-bound protein G magnetic beads at 4℃ for 1 h and washed with low salt reaction buffer and high salt reaction buffer. m^6^A-antibody-bound RNA was extracted from the Dynabeads using Buffer RLT (Qiagen, Hilden, German) and further incubated with Dynabeads MyOne Silane (Life Technologies, West Palm Beach, FL). The RNA and Dynabeads mixture were precipitated with 100% ethanol and washed with 70% ethanol, and then re-suspend with nuclease-free water. The supernatant was carefully collected after the beads were pulled to the side of the tube by a magnetic field. Real-time PCR was carried out following m^6^A-IP to quantify the changes to m^6^A methylation of a certain target gene. The sequences of primers are presented in Additional file 1: Table S1.

### Preparation of Gold nanocluster-assisted delivery of siRNA (GNC-siRNA) complex

The positively charged GNCs (1 μg/mL) were mixed with siRNA solution in ultrapure water, and shaken on a bench-top shaker for 1 h to complete the binding of siRNA onto the GNCs via electrostatic interaction. The METTL7B siRNA was added into the GNC solution in different concentrations to determine the saturated concentration of siRNA solution, with the weight ratio of siRNA to GNCs varied from 0:1 to 100:1. The prepared samples were abbreviated as GNC-siRNA. The process was performed according to the previous study [22].

### Statistical analysis

Statistical analyses were performed by GraphPad Prism 7.0 (GraphPad Software, La Jolla, CA, USA) for experimental analyses. The results are represented as the means ± SD of at least three independent experiments of biological replicates. Comparisons between two groups were analyzed by Student’s T-tests. One-way analysis of variance (ANOVA) followed by Dunnett’s test was used for comparisons among multiple groups. The relationship between METTL7B and SOD1, HMOX1, GPX4 expression levels was determined using Pearson correlation analysis. Differences were considered statistically significant when *P* < 0.05. Asterisks indicate statistical significance compared to the corresponding control: *, *P* < 0.05; **, *P* < 0.01; ***, *P* < 0.001 and ****, *P* < 0.0001.

## Results

### METTL7B was overexpressed in TKIs-resistant LUAD

**Fig. 1:**
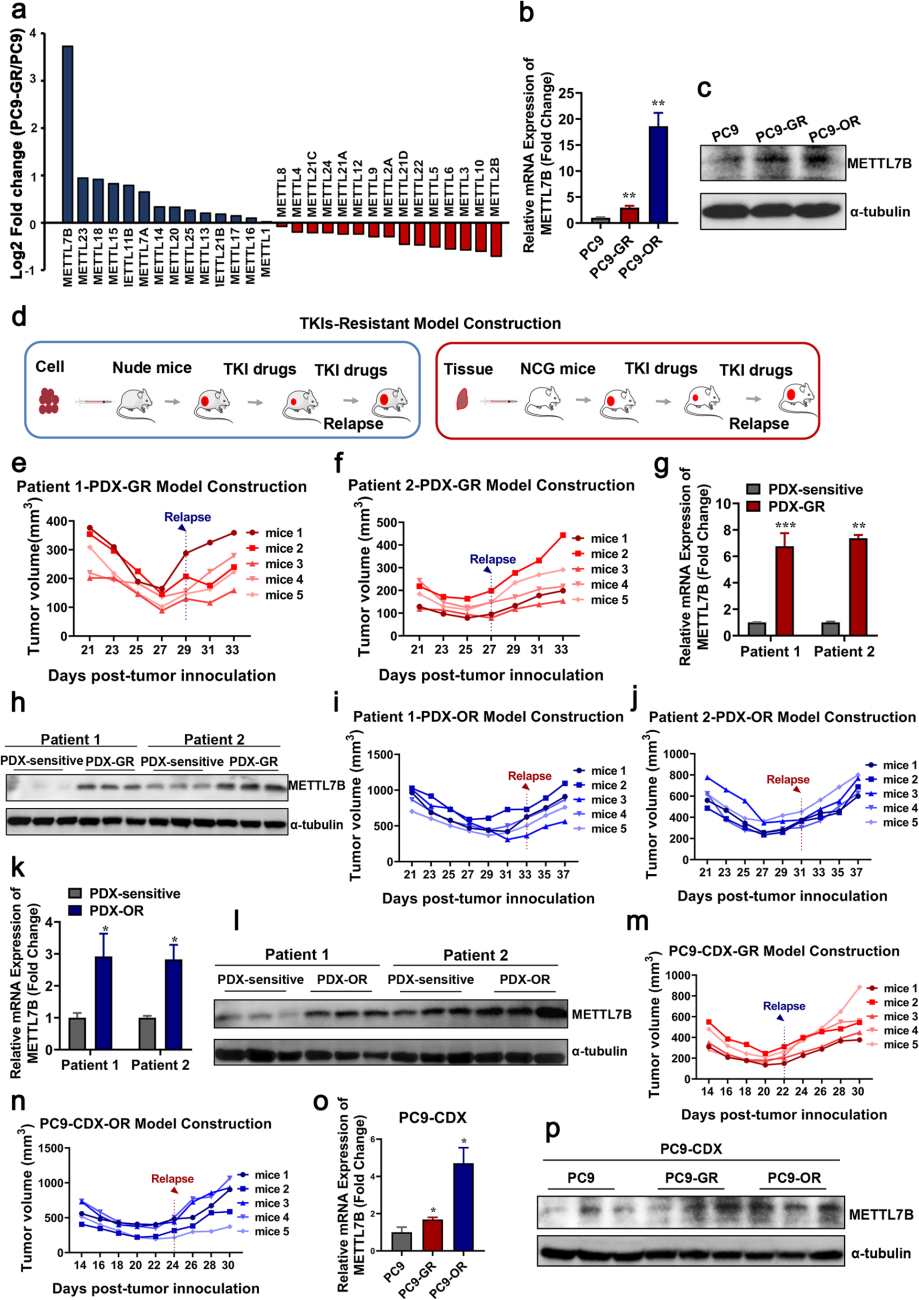
METTL7B was overexpressed in TKIs-resistant LUAD cells. **a** Bioinformatics analysis of the differential expression of METTL family between PC9 and PC9-GR cells from Gene Expression Omnibus (GEO) databases GSE74253. **b** and **c** qRT-PCR and immunoblot analysis on the expression of METTL7B in PC9, PC9-GR and PC9-OR cells. **d** The TKI-resistant PDX and CDX tumor models. **e-l** Patient-derived tumors were implanted into the flank of BALB/c nude mice to form subcutaneous tumors. The mice with subcutaneously implanted tumors were treated with gefitinib or osimertinib (30 mg/kg, qd, po). The tumor volumes were presented from five mice per group. At the end of experiment, the tumors were taken out and METTL7B mRNA and protein levels were evaluated. **m-p** The same experiments with (**e-l**) but in PC9 cell-derived xenograft tumors. Quantification of relative METTL7B mRNA levels from three independent experiments were shown. *P < 0.05, **P < 0.01, ***P < 0.001

Previously, we identified that METTL7B, a member of the METTL family characterized with methyltransferase domains, promoted cell growth and tumor progression in LUAD [17]. However, the role of METTL members in TKIs-resistant LUAD was not reported. Here, bioinformatics analysis of mRNA expression of METTL members in gefitinib-sensitive PC9 cells and gefitinib-resistant PC9-GR cells indicated that METTL7B was remarkably up-regulated in PC9-GR cells as compared with PC9 cells (Fig. 1a) [23]. We further validated the expression of METTL7B in PC9, PC9-GR and PC9-OR (osimertinib-resistant) cells by qRT-PCR and Western blot analysis. Consistent results to bio-informatics analysis were found in these experiments (Fig. 1b-c).

To explore the correlation between METTL7B expression and TKIs resistance in LUAD in vivo, both LUAD CDX and PDX TKIs-resistant mouse models were established (Fig. 1d-f, 1i-j, 1m-n and Additional file 4: Figure S1). Xenografts were dissected for gene expression analysis, and the results showed that METTL7B was significantly increased in both mRNA and protein level in gefitinib and osimertinib -resistant xenografts as compared with those in gefitinib and osimertinib -sensitive xenografts (Fig. 1g-h, k-l, o-p). These findings indicate that METTL7B is involved in EGFR-TKIs resistance in LUAD.

### METTL7B induced resistance to TKIs in LUAD

**Fig. 2:**
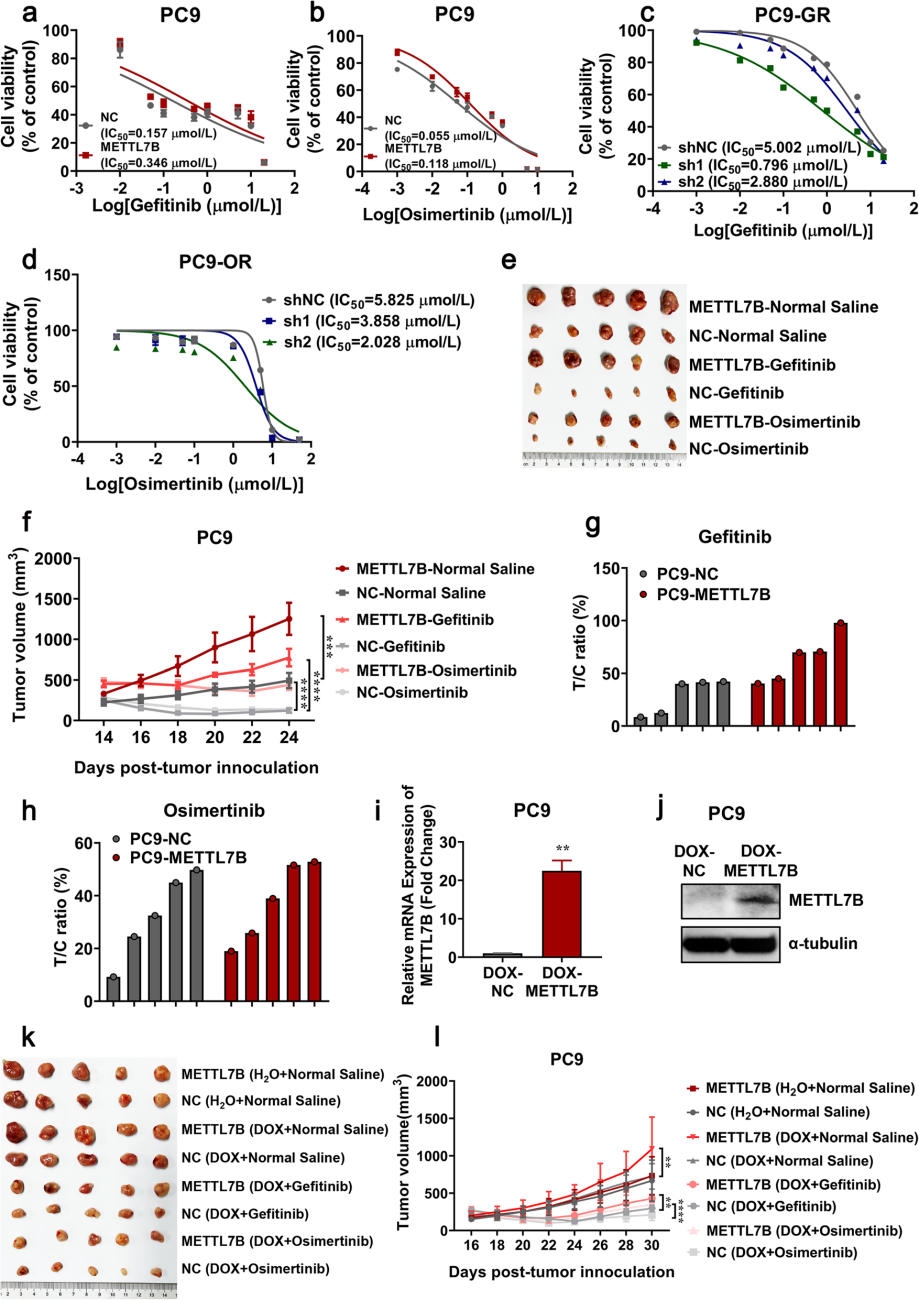
METTL7B induced resistance to TKIs in LUAD cells. **a** and **b** FLAG-NC and FLAG-METTL7B was stably transfected into TKIs-sensitive PC9 cell, and the cell viabilities were evaluated to measure IC_50_ of TKIs after treatment with different concentrations of gefitinib (**a**) and osimertinib (**b**) for 72 h. **c** and **d** shMETTL7B were stably transfected into gefitinib-resistant PC9-GR and osimertinib-resistant PC9-OR cells, and the cell viabilities were evaluated to measure IC_50_ of TKIs after incubation with different concentrations of gefitinib (**c**) and osimertinib (**d**) for 72 h. **e–h** PC9-FLAG-METTL7B or FLAG-NC were injected into the flank of BALB/c nude mice to form subcutaneous tumors. The tumors were treated with gefitinib and osimertinib every 2 d and the results were shown in tumor growth (**e**–**f**) and T/C ratio analysis (**g**-**h**) (*n* = 5 mice per group). T/C% = T_RTV_/C_RTV_ × 100%; T_RTV_, relative tumor volume after drug treatment; C_RTV_, relative tumor volume of vehicle control. **i**-**j** PC9-pInducer-METTL7B[Tet-on] cells were treated with or without DOX (1 μM) for 72 h. The expression levels of METTL7B were assayed by qRT-PCR (**i**) and Western blot (**j**). **k**-**l** PC9-pInducer-METTL7B[Tet-on] cells were injected into the flank of BALB/c nude mice to form subcutaneous tumors. The CDX-bearing mice (*n* = 5 mice per group) were treated with vehicle (normal saline), gefitinib (30 mg/kg, qd, po) or osimertinib (30 mg/kg, qd, po) and DOX (1.5 mg/mL). The growth of tumors was monitored every 2d. **P < 0.01, ***P < 0.001 and ****P < 0.0001

To investigate whether METTL7B could induce drug resistance in vitro, gene gain-of-function study was performed in LUAD cells. Cell viability analysis showed that the IC_50_ of TKIs (gefitinib and osimertinib) in METTL7B-overexpressed PC9 and HCC827 cells significantly increased by 2–3 folds, as compared with their vector control cells (Fig. 2a-b and Additional file 5: Figure S2a, c). These findings were further validated in METTL7B knock-down LUAD cells. The IC_50_ of gefitinib or osimertinib in METTL7B knock-down PC9-GR, H1975 and PC9-OR cells decreased to 40%—80%, as compared with their vector control cells (Fig. 2c-d and Additional file 5: Figure S2b-c).

To further investigate whether METTL7B could induce resistance to TKIs in LUAD in vivo, mice inoculated with either PC9 or METTL7B-overexpressed PC9 cells were treated with gefitinib, osimertinib (30 mg/kg) or vehicles. In the three groups treated with vehicles, the tumor volumes in METTL7B-overexpressed PC9 cells group were significantly increased as compared with those in vector control, which was consistent with our previous study [18] (Fig. 2e-h). With the treatment of gefitinib and osimertinib, the growth of tumors with ectopic expression of METTL7B still showed a notable rise (Fig. 2f), while the sizes of tumors were significantly decreased in vector control group, indicated that METTL7B could induce TKIs resistance in LUAD in vivo. In order to further rule out METTL7B-induced TKIs resistance due to cell growth, we constructed the doxycycline-inducible METTL7B expression system and monitored the sensitivity of tumors to gefitinib and osimertinib in LUAD in vivo. Consistent with the results in Fig. 2e-h, under the treatment of gefitinib and osimertinib, the tumor volumes in dox-inducible METTL7B group were significantly increased as compared with those in vector control (Fig. 2i-l). Overall, these findings suggest that METTL7B can induce TKIs resistance in LUAD cells both in vitro and in vivo*.*

### METTL7B promoted glutathione metabolism in LUAD cells

**Fig. 3:**
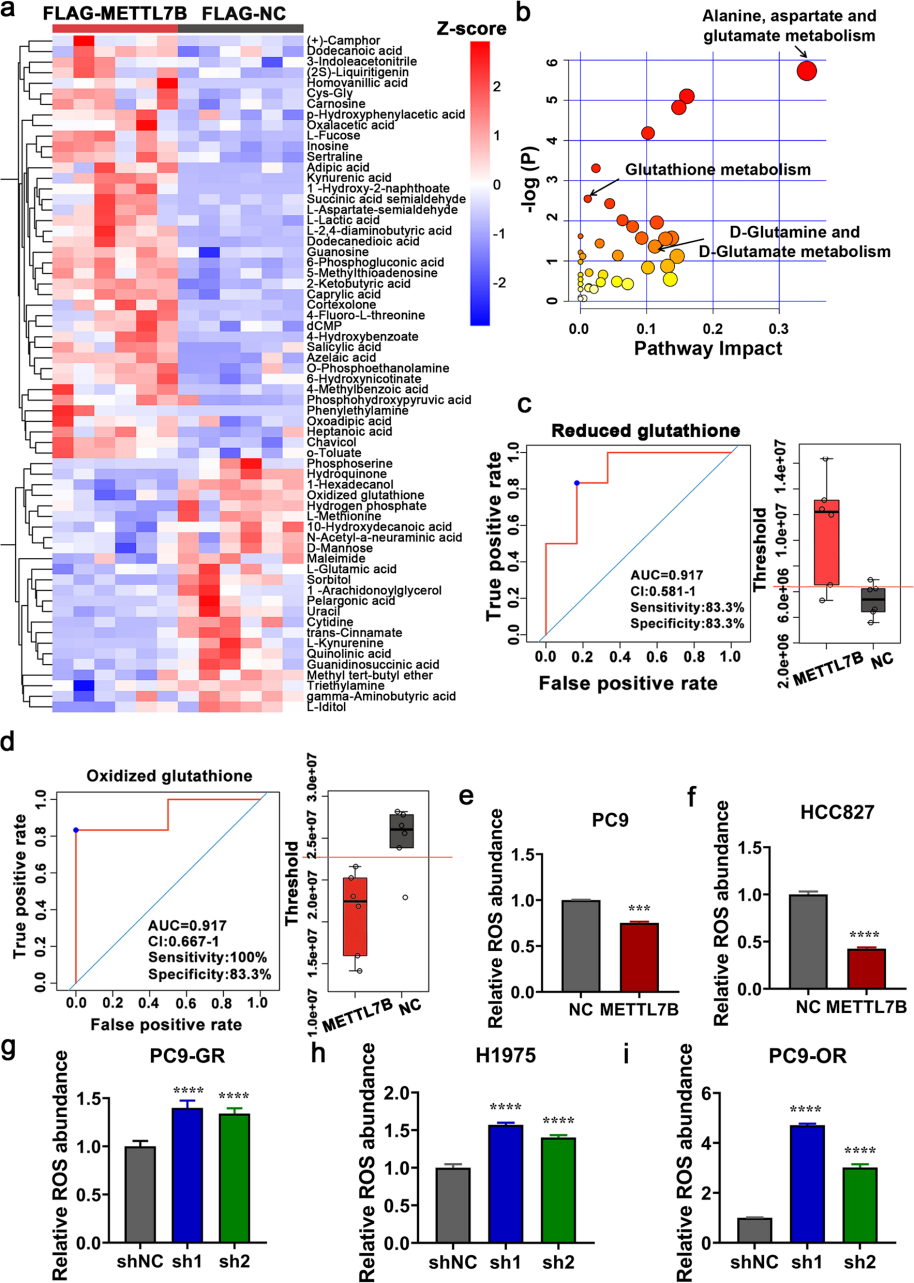
METTL7B promoted glutathione metabolism in LUAD cells. Untargeted metabolomic analysis by UPLC-MS/MS was detected in PC9 cells stably transfected with FLAG-NC or FLAG-METTL7B. **a** Heat map of differential metabolites. **b** KEGG pathway of differential metabolites. **c** and **d** ROC analysis for reduced glutathione and oxidized glutathione. **e**-**i** ROS level was detected in METTL7B-overexpressed PC9, HCC827 and METTL7B-suppressed PC9-GR, H1975 and PC9-OR cells. ***P < 0.001 and ****P < 0.0001

To systematically decipher the mechanism of METTL7B induced TKIs resistance in LUAD cells, un-targeted metabolomic analysis was performed in PC9 cells. Forty significantly upregulated and twenty-four significantly downregulated metabolites were found in METTL7B-overexpressed PC9 cells (Fig. 3a and Additional file 2: Table S2). KEGG pathway analysis showed that the differential metabolites were enriched in glutathione metabolism-related process, indicating METTL7B was involved in ROS-scavenging pathways (Fig. 3b and Additional file 3: Table S3). The receiver operating characteristic (ROC) analysis indicated that reduced glutathione was increased and oxidized glutathione was decreased in METTL7B-overexpressed PC9 cells as compared with those in vector control cells (Fig. 3c-d). These findings were further validated by examining the activity of GSH and GSSG in METTL7B-overexpressed PC9 cells. Our data showed that overexpression of METTL7B could increase the GSH activity and decreased the GSSG activity in PC9 cells, which was consistent with the results of metabolomic analysis (Additional file 6: Figure S3a-b). The glutathione metabolism-related process included ROS-scavenging pathways and RNS-scavenging pathways. The results of ROS and RNS detection indicated that METTL7B could decrease the levels of intracellular ROS and RNS (Fig. 3e-i and Additional file 6: Figure S3c-g). These findings indicate that METTLB promoted the glutathione metabolism in LUAD cells.

### METTL7B promoted ROS scavenging through upregulation of antioxidant enzymes

**Fig. 4:**
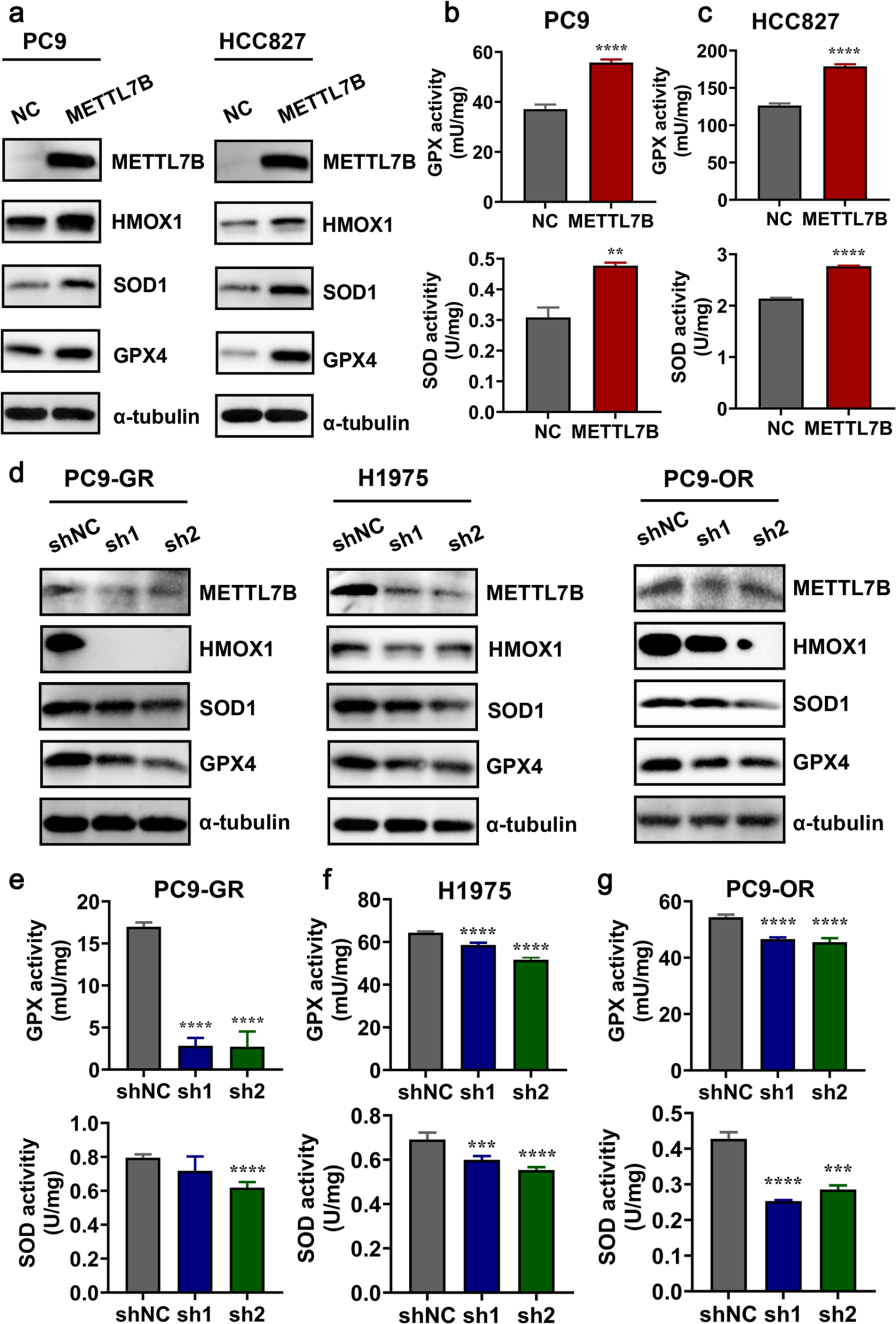
METTL7B enhanced ROS scavenging through upregulation of antioxidant enzymes. **a** FLAG-NC and FLAG-METTL7B was stably transfected into TKIs-sensitive PC9 and HCC827 cells, and relative protein levels were measured by Western blot. **b** and **c** The enzymatic activities of GPX and SOD were detected by Cu/Zn-SOD and Mn-SOD Assay Kit with WST-8 and Cellular Glutathione Peroxidase Assay Kit with NADPH in METTL7B-overexpressed PC9 (**b**) and HCC827 cells (**c**). **d** The same experiments with (**a**) but in METTL7B-suppressed PC9-GR, H1975 and PC9-OR cells. **e–g** The same experiments with (**b-c**) but shMETTL7B were stably transfected into gefitinib-resistant PC9-GR, H1975 and osimertinib-resistant PC9-OR cells. **P < 0.01, ***P < 0.001 and ****P < 0.0001

Given the functional role of METTLB in promoting glutathione metabolism, its underlying molecular mechanism was further examined both in vitro and in vivo. Glutathione (GSH), a tripeptide thiol antioxidant composed of the amino acids glutamic acid, cysteine, and glycine, is the main ROS scavenger in cells and plays a central role in ROS-induced redox signaling [24, 25]. The protein expression of key enzymes that regulating ROS scavenging indicated that only GPX4, SOD1 and HMOX1 were significantly up-regulated in METTL7B-overexpressed PC9 and HCC827 cells as compared with vector control cells (Fig. 4a and Additional file 7: Figure S4a-b). Increased enzymatic activities of GPX and SOD were detected in METTL7B-overexpressed PC9 and HCC827 cells as compared with vector control cells (Fig. 4b-c). Downregulated expressions of GPX4, SOD1 and HMOX1 were further validated by shRNA knock-down of METTL7B in PC9-GR, H1975 and PC9-OR cells (Fig. 4d and Additional file 7: Figure S4c-e). Similarly, the enzymatic activities were found decreased in METTL7B-suppressed PC9-GR, H1975 and PC9-OR cells, respectively (Fig. 4e-g).

**Fig. 5:**
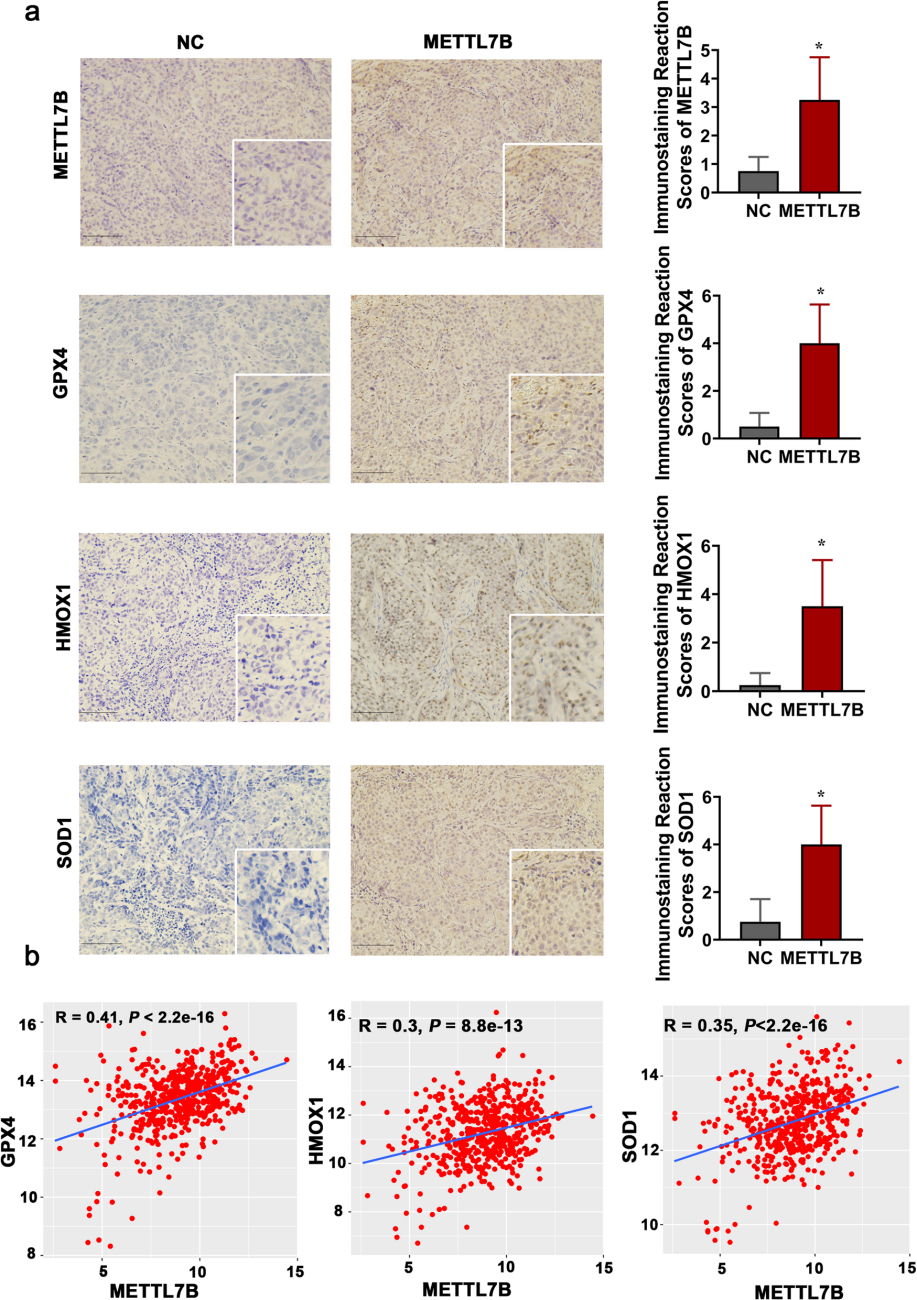
The expression of METTL7B was positively correlated with GPX4, SOD1 and HMOX1. **a** The tumors in Fig. 2e were used to measure the expressions of METTL7B, GPX4, SOD1 and HMOX1 by immunohistochemistry. Scale bar: 100 μm. **b** The correlation between METTL7B and GPX4, SOD1 and HMOX1 in LUAD tissues from TCGA database. *P < 0.05

These in vitro results were verified in LUAD CDX mouse model (Fig. 2e). Immunohistochemistry assay identified upregulated protein expression of METTL7B, GPX4, SOD1 and HMOX1 in METTL7B-overexpressed PC9 cell-derived tumors (Fig. 5a). Moreover, analysis of the expression data from TCGA database indicated that the expression of METTL7B was positively correlated with these three key ROS scavengers [GPX4 (*R* = 0.41, *P* < 2.2e-16), HMOX1 (*R* = 0.3, *P* < 8.8e-13), and SOD1 (*R* = 0.35, *P* < 2.2e-16)] (Fig. 5b). Our results indicate that METTL7B can accelerate ROS scavenging through upregulation of antioxidant enzymes.

### METTL7B induced TKIs resistance in LUAD cells in a ROS-scavenging-dependent manner

**Fig. 6:**
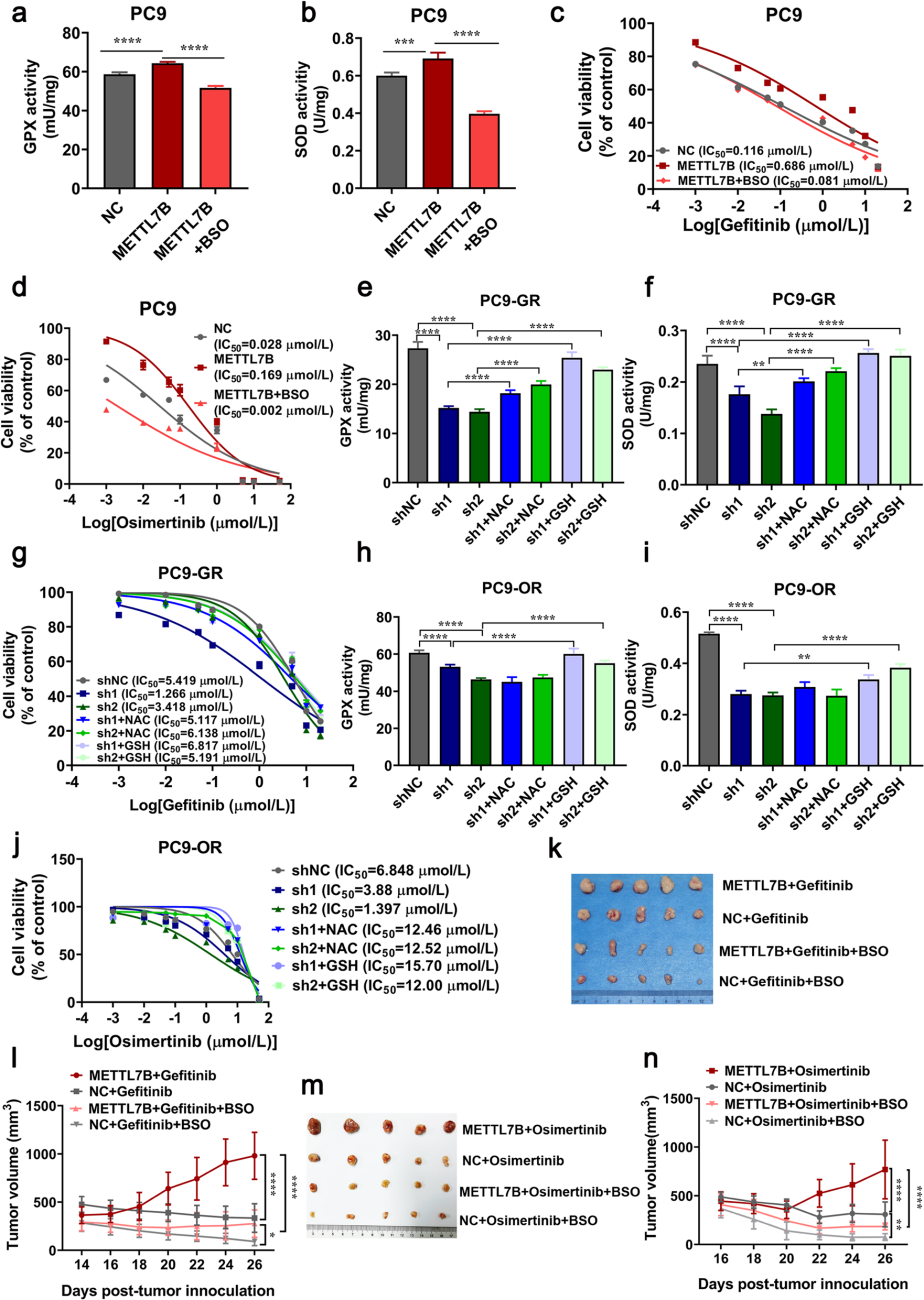
METTL7B-induced TKIs resistance was associated with ROS scavenging in LUAD cells. **a-d** FLAG-NC and FLAG-METTL7B was stably transfected into gefitinib-sensitive PC9 cells. The enzymatic activities of GPX (**a**) and SOD (**b**) were measured by oxidative stress assay kits with or without BSO (200 μM for 24 h). Cell viability was detected 72 h after treatment of different concentrations of gefitinib (**c**) and osimertinib (**d**). **e-j** shMETTL7Bs were stably transfected into gefitinib-resistant PC9-GR and osimertinib-resistant PC9-OR cells. The enzymatic activities of GPX (**e** and **h**) and SOD (**f** and **i**) were measured by oxidative stress assay kits with or without NAC (10 mM for 6 h) or GSH (8 mM for 6 h). Cell viability was detected 72 h after treatment of different concentrations of gefitinib (**g**) and osimertinib (**j**). **k-n** PC9 cells with FLAG-METTL7B or FLAG-NC were injected into the flank of BALB/c nude mice to form subcutaneous tumors. The mice with subcutaneously implanted tumors were treated with TKIs (gefitinib and osimertinib) (30 mg/kg, qd, po) or combinations of TKIs and BSO (450 mg/kg, qod, ip) as indicated. The growth of tumors was monitored every 2 d. Tumor volume and body weights were presented as mean ± SD from five mice per group. *P < 0.05, **P < 0.01, ***P < 0.001 and ****P < 0.0001

Given that METTL7B could increase the enzymatic activity of antioxidants in LUAD, we further evaluated whether METTL7B-induced TKIs resistance was antioxidant- dependent. Buthionine sulphoximine (BSO), a specific inhibitor of ROS scavenging, was used in METTL7B-overexpressed PC9 cells. The results showed BSO treatment (200 μM) re-sensitized the METTL7B-overexpressed PC9 cells to gefitinib and osimertinib, accompanied with the decreased enzymatic activities of GPX and SOD (Fig. 6a-d). Moreover, the increased sensitivity toward gefitinib and osimertinib and reduced enzymatic activities of GPX and SOD induced by knock-down of METTL7B in PC9-GR and PC9-OR cells could be reversed by ROS scavengers, either N-acetyl-L-cysteine (NAC) (10 mM) or antioxidant GSH (8 mM) (Fig. 6e-j). Consistent results were found in both METTL7B-overexpressed HCC827 cells and METTL7B-suppressed H1975 cells (Additional file 8: Figure S5a-g).

To verify these findings in vivo, we examined whether activating the ROS system, by suppressing GSH biosynthesis with BSO, could abrogate METTL7B-induced gefitinib and osimertinib resistance in LUAD CDX mouse model. It was found that overexpression of METTL7B conferred resistance to gefitinib and osimertinib, and the effect was abrogated by BSO treatment in these mouse model (Fig. 6k-n). These findings suggest that METTL7B can induce TKIs resistance in LUAD cells in a ROS-scavenging dependent manner.

### METTL7B upregulated the protein expression of ROS-scavenging genes mediated by m^6^A modification.

**Fig. 7:**
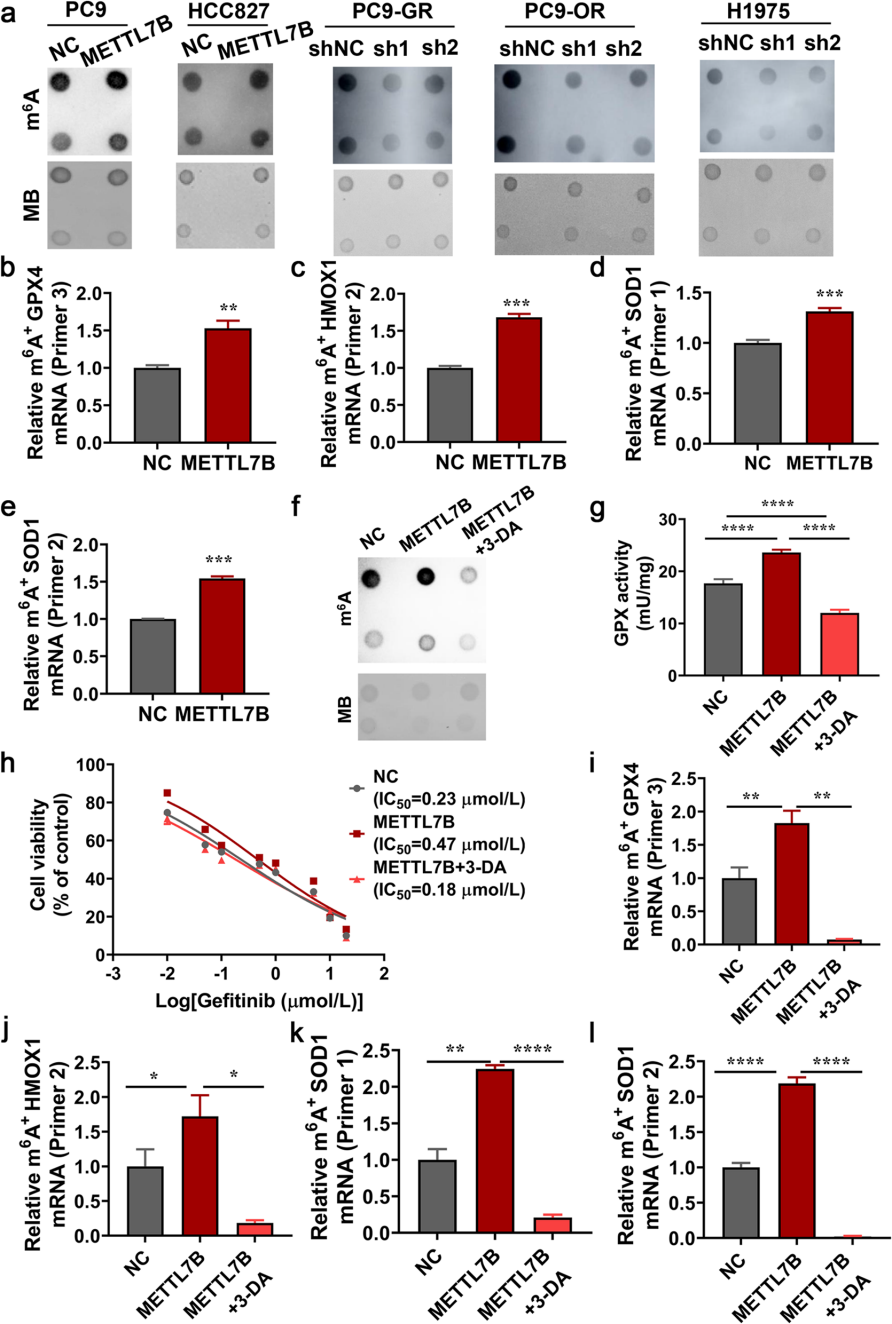
METTL7B regulated the stability of ROS-scavenging related genes mediated by m^6^A modification. **a** RNA dot blot analysis (upper panel) of m^6^A levels in METTL7B-overexpressed PC9, HCC827 and METTL7B-suppressed PC9-GR, H1975 and PC9-OR cells. Methylene blue staining served as a loading control (lower panel). **b**-**e** m^6^A immunoprecipitation and qRT-PCR performed to determine the change of GPX4, HMOX1 and SOD1 mRNA with m^6^A modification. **f-l** Overexpression of METTL7B in PC9 cells upregulated m^6^A levels, GPX and SOD activity, with the increased IC_50_ to gefitinib, and the effect was reversed by 3-DA (10 μM for 72 h). *P < 0.05, **P < 0.01, ***P < 0.001 and ****P < 0.0001

The METTL7B is consisted of nuclear export signal (NES), methyltransferase domain, and SAM binding motif I (GXGXG) (Additional file 9: Figure S6a) [26, 27]. We next explored whether METTL7B modulated ROS related genes by m^6^A modification. The m^6^A MS analysis indicated that m^6^A level was significantly increased in METTL7B-overexpressed PC9 cells as compared with vector control cells (Additional file 9: Figure S6b). The levels of m^6^A modification were significantly increased in METTL7B-overexpressed PC9 and HCC827 cells as compared with vector control cells as shown by the m^6^A-specific antibody dot blot (Fig. 7a). Moreover, knock-down of METTL7B by shRNA significantly reduced m^6^A modification level in PC9-GR, H1975 and PC9-OR cells (Fig. 7a).

Then, we detected the m^6^A modification in m^6^A consensus sequence within CDS and the 3’ UTR of GPX4, SOD1 and HMOX1 mRNA (Additional file 9: Figure S6c). The results revealed a higher m^6^A modification in the 3’ UTR of GPX4, the CDS of SOD1 and the 3’ UTR of HMOX1 mRNA in METTL7B-overexpressed PC9 cells (Fig. 7b-e). In order to further validate these findings, 3-Deazadenosine (3-DA), a S-adenosylmethionine inhibitor which could deplete the m^6^A modification on mRNA [28], was applied. Treatment of 3-DA (10 μM) reduced the level of m^6^A modification induced by METTL7B overexpression in PC9 cells (Fig. 7f). Furthermore, increased GPX activity, insensitivity to gefitinib and the mRNA m^6^A modification of antioxidant genes induced by overexpression of METTL7B were all abrogated by the treatment of 3-DA in PC9 cells (Fig. 7g-l). Taken together, our results suggest that METTL7B can enhance the protein expression of ROS-scavenger genes in LUAD cells through m^6^A modification eventually leading to TKI resistance.

### Knock-down of METTL7B reversed the resistance toward gefitinib and osimertinib in LUAD both in vitro and in vivo.

**Fig. 8:**
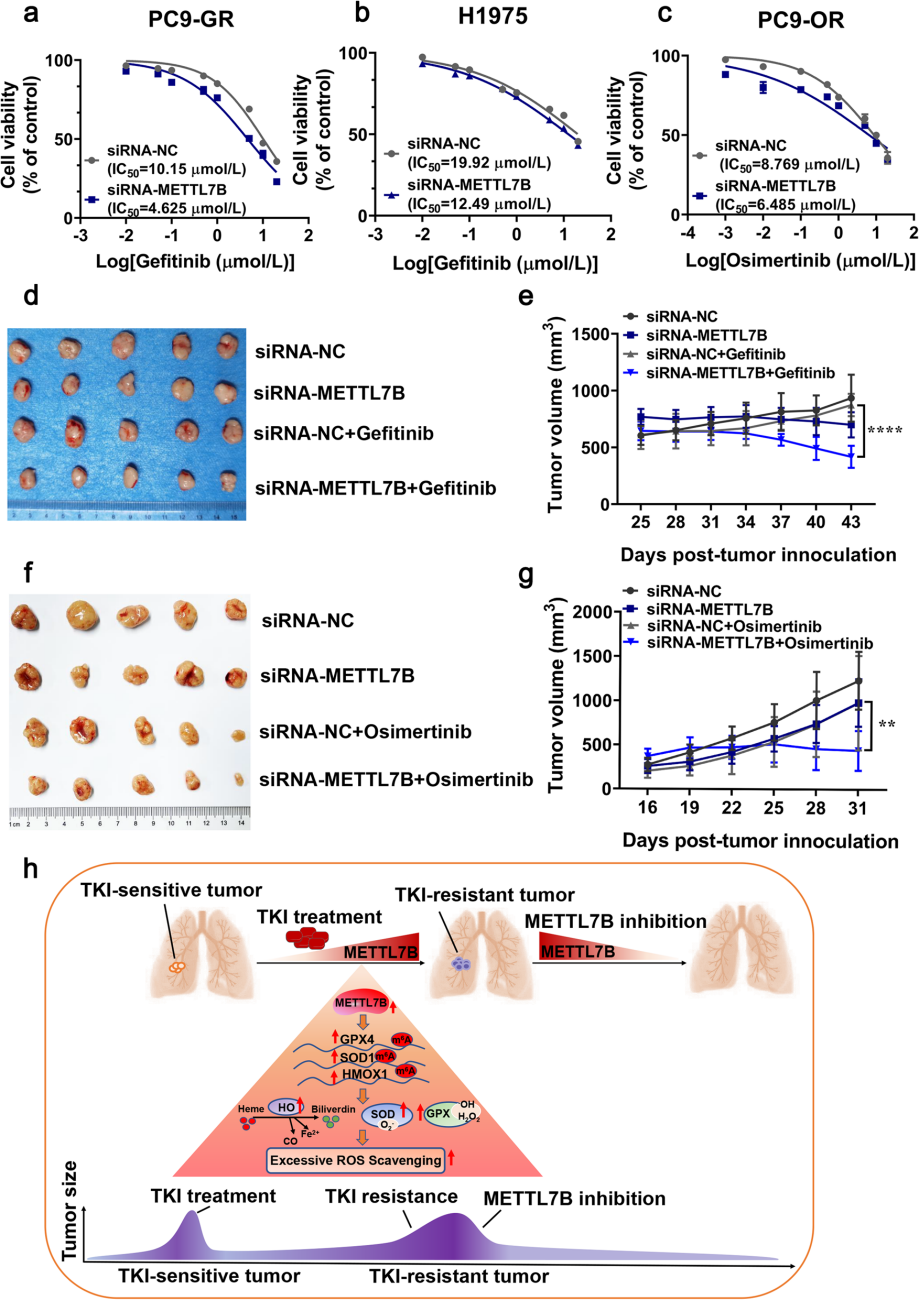
Knock-down of METTL7B reversed the resistance toward TKIs in LUAD cells both in vitro and in vivo. **a**-**c** Gold nanocluster-assisted delivery of METTL7B-siRNA was transfected into gefitinib-resistant PC9-GR, H1975 and osimertinib-resistant PC9-OR cells, and the cell viability was evaluated to measure IC_50_ of TKIs after treatment with different concentrations of gefitinib and osimertinib for 72 h. **d-g** PC9-GR (d-e) and PC9-OR (f-g) cells were injected into the two flanks of BALB/c nude mice to form subcutaneous tumors. The mice with subcutaneously implanted tumors were treated with TKIs (gefitinib and osimertinib) (30 mg/kg, qd, po) and GNC-siNC or GNC-siMETTL7B as indicated. The growth of tumors was monitored every 3 d. Tumor volume and body weights were presented as mean ± SD from five mice per group. **h** The schema represented the critical link between METTL7B, GPX4, HMOX1 and SOD1 in TKIs-resistant LUAD cells. **P < 0.01 and ****P < 0.0001

To examine the potential significance of targeting METTL7B in reversing EGFR‐TKIs resistance in LUAD, gold nanocluster-assisted delivery of siRNA was used for both in vitro and in vivo cell growth assay [22]. Gefitinib-resistant PC9-GR and H1975 cells, and osimertinib-resistant PC9-OR cells were treated with the well-characterized GNC-siMETTL7B (Additional file 10: Figure S7) as well as vector control in combination with gefitinib or osimertinib. The results showed that combination of siRNA-METTL7B and gefitinib significantly re-sensitized PC9-GR and H1975 cells to gefitinib with a much lower IC_50_ as compared with that of vector control (Fig. 8a-b). Similar results were found in METTL7B-suppressed PC9-OR cell (Fig. 8c).

Further investigation was performed to examine the effects of GNC-siMETTL7B in LUAD CDX mouse model. Coincided with the in vitro results, combination treatment with GNC-siMETTL7B (6 mg siRNA per mouse equivalent) significantly suppressed tumor growth as compared with treatment of gefitinib or osimertinib (30 mg/kg) alone (Fig. 8d-g). These findings demonstrate that METTL7B could be a potential therapeutic target for reversing TKIs resistance in LUAD.

## Discussion

Previously, our group and others had revealed that increased expression of METTL7B contributed to cancer cell proliferation, migration, and invasion, resulting in advanced stages of tumor development and poor survival [18–20]. Here, we found that METTL7B promoted TKIs resistance in LUAD via accelerating the scavenging of ROS. Mechanistically, METTL7B upregulated the protein expression of antioxidant genes via m^6^A modification. In addition, targeting METTL7B by GNC-siMETTL7B re-sensitized LUAD cells to TKIs both in vitro and in vivo. These results indicated that METTL7B could be a promising therapeutic target for reversing TKIs resistance in LUAD.

Intra-tumoral hypoxia leads to abnormal metabolism and chromosome instability in cancer cells, which results in excessive ROS generation in tumor microenvironment [11]. In order to maintain redox-homeostasis, tumor cells increase enzymatic activity of antioxidants and activate enzyme-independent pathways to scavenge ROS [29]. In fact, a number of studies have shown that most of the anti-tumor therapies including chemotherapy [30], radiotherapy [31], immunotherapy [32] induce ROS production and activate death signaling cascades in cancer cells. Because METTL7B enhanced the expression of essential ROS-scavenging enzymes, SOD1, GPX4 and HMOX1 as well as their enzymatic activities [33–35], it is worthy to explore whether METTL7B could promote resistance to other anti-cancer reagents in addition to TKIs in the future.

Drug resistance caused by EGFR mutations has been a major issue of antineoplastic therapy failure in using EGFR inhibitors [5]. The contribution of EGFR-independent molecules and pathways to TKIs resistance has attracted attention [8–10]. A weak correlation between the protein expression of METTL7B and EGFR was found in LUAD tissue microarray [18], which promoted us to explore the role of METTL7B in TKIs resistance in LUAD. However, no significant change was found in the protein expression as well as phosphorylation of EGFR in both METTLB-overexpressed and -suppressed LUAD cells (data not shown). Although METTL7B did not directly regulate the activation of EGFR signaling pathway, it enhanced the ROS scavenging signaling pathway by upregulating the expression of antioxidant enzymes, indicating that METTL7B induced TKIs resistance in an EGFR-independent manner (Fig. 4). By using GNC-siRNA targeting METTL7B, the gefitinib and osimertinib resistance was reversed in vivo. Notably, we also identified low expression of METTL7B in low-grade LUAD and tumor adjacent normal tissues (data not shown). These findings provide a rationale for combined use of METTL7B inhibitors with TKIs in patients with EGFR-independent TKIs resistance.

In this study, we identified three downstream targets of METTL7B, GPX4, SOD1 and HMOX1. GPX4 is a critical inhibitor of ferroptosis, a process during which iron-induced peroxidation of membrane lipids causes programmed cell death that distinct from apoptosis [36]. SOD1 is a well-known enzyme for defending against oxidative stress by catalyzing the disproportionation of superoxide into H_2_O_2_ and O_2_ [33]. HMOX1 is an anti-oxidant, anti-inflammatory and anti-apoptotic protein which promotes metabolic reprogramming and antioxidant defense [37]. Previous studies have shown that Kelch-like ECH-associated protein 1 (KEAP1)-nuclear factor (erythroid-derived 2)-like 2 (NRF2) antioxidative pathway is involved in the process of drug resistance in tumors [38, 39]. Once Oxidative stress commonly induced the conformation of KEAP1, which led to dissociation of NRF2. The dissociated NRF2 entered the nucleus, leading to upregulation of the expressions of ROS metabolic genes, such as GPX4, SOD1, NQO1 and HMOX1 [38, 39]. However, we identified that the expression as well as nuclear-cytoplasmic distribution of NRF2 were not changed in METTL7B inducted PC9 cells (Additional file 11: Figure S8), indicating that METTL7B regulated ROS metabolism in an NRF2-independent manner. In this study, we uncovered a novel role of METTL7B in metabolic regulation based on the findings that METTL7B regulated antioxidant genes.

METTL7B was found to be involved in cell growth among various malignant tumors [18–20]. However, its underlying regulatory mechanism remains unclear. Our data suggested for the first time that METTL7B played a role in m^6^A modification of mRNAs. The level of m^6^A modification was elevated in METTL7B-overexpressed PC9 and HCC827 cells and knock-down of METTL7B significantly reduced the levels of m^6^A modification. The status of m^6^A modification of mRNA in the antioxidant genes (GPX4, SOD1 and HMOX1) was directly regulated by METTL7B, and the effect was eliminated by m^6^A modification inhibitor. Recent studies showed that the m^6^A readers, such as IGF2BPs or RNA-binding protein HNRNPD, could selectively bind to the target mRNA and promote the mRNA stability. In gastric cancer, METTL3 promoted the m^6^A modification of HDGF mRNA, and the m^6^A reader IGF2BP3 directly recognized and bound to the m^6^A site on HDGF mRNA and enhanced HDGF mRNA stability [40]. Another study in H/R-treated cardiomyocytes revealed that METTL3 methylated the 3’ UTR of TFEB mRNA, thereby promoting the association of HNRNPD with TFEB pre-mRNA and decreasing the mRNA level of TFEB [41]. Therefore, more physiological target genes of METTL7B and the interaction with m^6^A readers warranted further exploration.

Previous studies had showed the strong correlation between m^6^A modification and drug resistance. The m^6^A demethylase FTO was overexpressed in leukemia cells, which enhanced mRNA stability of proliferation/survival transcripts, increased protein synthesis and induced TKIs resistance [42]. m^6^A reader IGF2BP2 bound to CDS region of ERBB2 mRNA via m^6^A modification, increased ERBB2 translation efficacy, and induced acquired resistance to TKIs [43]. METTL3 could promote m^6^A modification and translation of YAP mRNA through recruiting YTHDF1/3 and eIF3b and increased mRNA stability of YAP via the MALAT1-miR-1914-3p-YAP axis, thereby inducing cisplatin resistance in NSCLC [44]. Hypoxia-induced YTHDF1 upregulated the expression of KEAP1 via m^6^A modification and induced the sensitivity to cisplatin via inhibiting drug-resistant-associated gene AKR1C1 [45]. In sorafenib-resistant hepatocellular carcinoma, METTL3 was significantly down-regulated and could promote m^6^A modification of FOXO3 3’UTR increasing its stability via recruiting YTHDF1, thereby enhancing sorafenib resistance of HCC [46]. In this paper, we found that METTL7B could promote TKIs resistance in NSCLC. Further mechanism revealed that METTL7B promoted expressions of SOD1, HMOX1 and GPX4 via m^6^A modification, indicating that METTL7B may exercise m^6^A function acting as the RNA methyltransferase. And more biochemical experiments of METTL7B require further verification in the future.

## Conclusions

In the present study we uncovered a novel role of METTL7B in promoting TKIs resistance in LUAD. Under the surviving stress upon TKIs, elevated METTL7B in LUAD cells accelerated the scavenging of excessive ROS in tumor microenvironment by upregulating the protein levels and enzymatic activities of three antioxidant enzymes GPX4, SOD1 and HMOX1 by m^6^A modification in their mRNAs (Fig. 8h). Our study provides new insights that METTL7B could be a potential therapeutic target for reversing TKIs resistance in LUAD.

## Supplementary Information

**Additional file 1:** **Table S1.** Primers used for real-time RT-PCR.

**Additional file 2:** **Table S2.** The differential metabolites in PC9 cells stably transfected with FLAG-NC (PC9-NC) or FLAG-METTL7B (PC9-7B).

**Additional file 3: Table S3.** KEGG pathway of differential metabolites in PC9 cells stably transfected with PC9-NC or PC9- METTL7B.

**Additional file 4:** **Figure S1.** Pathological information of LUAD patients for PDX model construction. **a** HE and immunohistochemical staining (CK7, TTF1, NAPSINA, CK5/6, P63, SYN and P40) on one LUAD patient tissue sections. **b** The mutation sites in tumor tissue of the LUAD patient in **a**. **c** HE staining on the other LUAD patient tissue section. **d** The information of EGFR mutation in tumor tissue of the LUAD patient in **c**.

**Additional file 5: Figure S2.** The IC_50_ of TKIs in METTL7B-overexpressed HCC827 and METTL7B-suppressed H1975 cells. **a** FLAG-NC and FLAG-METTL7B was stably transfected into TKIs-sensitive HCC827 cell, and the cell viabilities were evaluated to measure IC_50_ of TKIs after treatment with different concentrations of gefitinib and osimertinib for 72 h. **b** METTL7B-shRNAs were stably transfected into gefitinib-resistant H1975 cell, and the cell viability was evaluated to measure IC_50_ of gefitinib after treatment with different concentrations of gefitinib for 72 h. **c** The statistical results of IC_50_ in METTL7B-overexpressed PC9, HCC827 and METTL7B-suppressed PC9-GR, H1975 and PC9-OR cells. *P < 0.05, **P < 0.01 and ****P < 0.0001.

**Additional file 6:** **Figure S3.** METTL7B promoted glutathione metabolism in LUAD cells. **a** and **b** GSH and GSSG levels were detected in PC9 cells stably transfected with FLAG-NC or FLAG-METTL7B. **c-g** RNS level was detected in METTL7B-overexpressed PC9 (**c**), HCC827 (**d**) and METTL7B-suppressed PC9-GR (**e**), H1975 (**f**) and PC9-OR (**g**) cells. ***P < 0.001 and ****P < 0.0001.

**Additional file 7:** **Figure S4.** METTL7B didn’t changed expressions of other antioxidant enzymes. PC9 (**a**) and HCC827 (**b**) cells were stably transfected with FLAG-NC or FLAG-METTL7B and the protein levels of SOD2, GPX1, GPX2, GPX7 and GPX8 were measured by Western blot. **c-e** The same experiment of (**a-b**) but in METTL7B-suppressed PC9-GR (**c**), H1975 (**d**) and PC9-OR (**e**) cells.

**Additional file 8:** **Figure S5.** METTL7B-induced gefitinib resistance was associated with ROS scavenging in LUAD cells. The same experiment of Fig. 6 but in METTL7B-overexpressed HCC827 (**a-d**) and METTL7B-suppressed H1975 cells (**e-g**). *P < 0.05, ***P < 0.001 and ****P < 0.0001.

**Additional file 9:** **Figure S6.** METTL7B regulated the stability of ROS-scavenging related genes mediated by m^6^A modification. **a** The protein domains of METTL7B. **b** LC-MS/MS quantification of the m^6^A/A in mRNA of NC and METTL7B-overexpressed PC9 cell. **c** Sequence analysis of m^6^A consensus sequence in GPX4, HMOX1 and SOD1 from SRAMP website. The primers for MeRIP-qPCR were presented in Additional file 1: Table S1. ***P < 0.001.

**Additional file 10:** **Figure S7.** The characterizations of GNC-siRNA. **a** and **b** Characteristics of GNC-siRNA-NC and siRNA-METTL7B complex. **c-e** Excitation and emission spectrum of GNCs, GNC-siRNA-NC and GNC-siRNA-METTL7B. **f** Diameter of the prepared GNCs and GNC-siRNA complex.

**Additional file 11:** **Figure S8.** The expression of NRF2 was not changed by overexpression of METTL7B. **a** and **b** FLAG-NC, FLAG-METTL7B (**a**) or shNC, shMETTL7Bs (**b**) were stably transfected into PC9 cells or PC9-GR cells, and NRF2 protein levels were measured by Western blot. b Nucleocytoplasmic distribution of METTL7B in PC9 cells stably transfected with FLAG-NC or FLAG-METTL7B.

## Data Availability

The datasets used and/or analyzed during the current study are available from the corresponding author on reasonable request.

## Abbreviations

METTL7B: Methyltransferase like 7B
LUAD: Lung adenocarcinoma
EGFR: Epidermal growth factor receptor
TKIs: Tyrosine kinase inhibitors
CDX: Cell-Derived tumor Xenograft
PDX: Patient-Derived tumor Xenograft
BSO: Buthionine sulphoximine
NAC: N-acetyl-L-cysteine
GSH: Glutathione
GNC: Gold nanocluster
