# Supplementary figures and images for: Methyltransferase like 7B is a potential therapeutic target for reversing EGFR-TKIs resistance in lung adenocarcinoma
Source: Mol Cancer. 2022 Feb 10;21:43. doi: 10.1186/s12943-022-01519-7 (PMC8830004; doi:10.1186/s12943-022-01519-7)

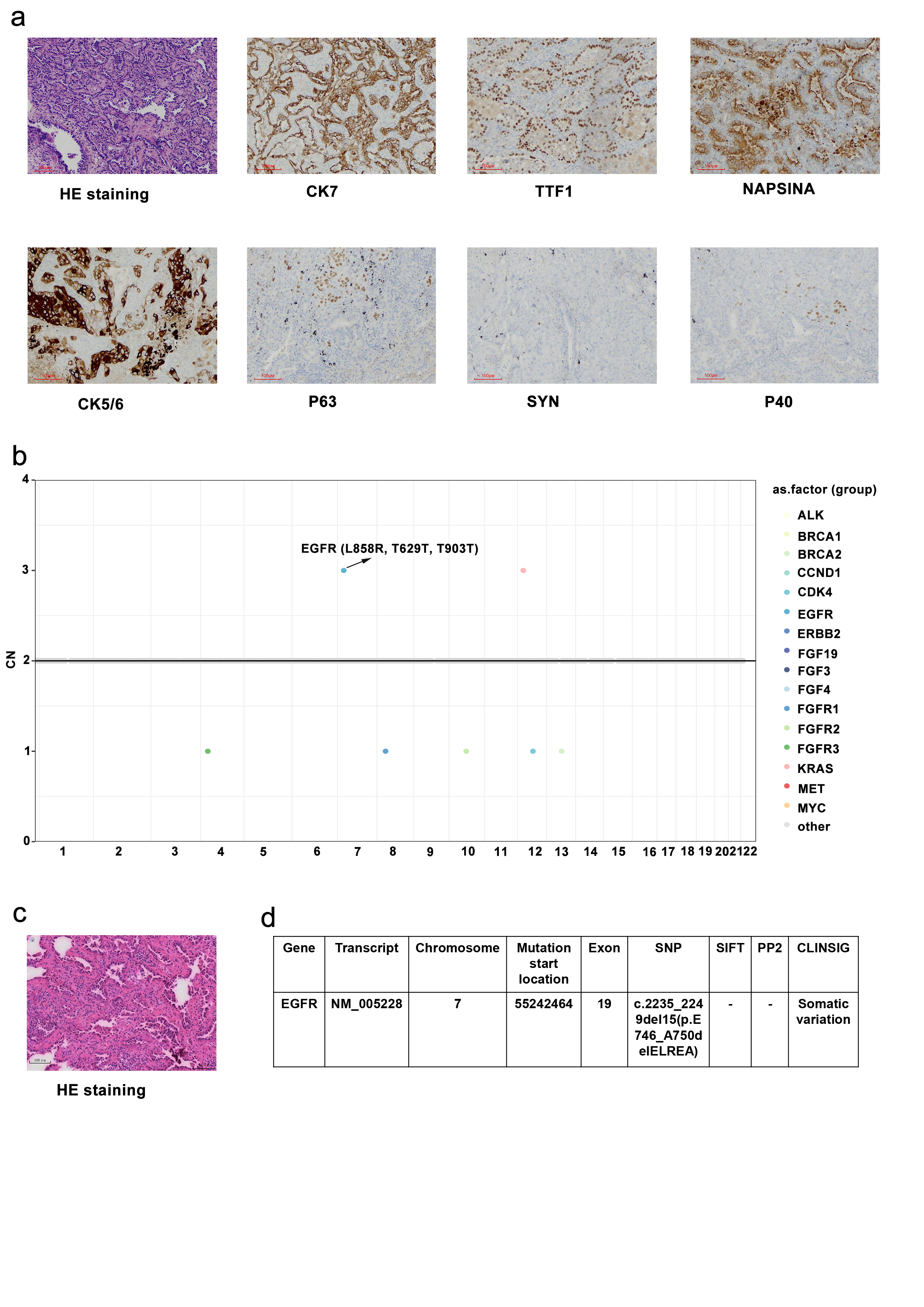

Supplement: Supplementary file 4 — Additional file 4: Figure S1. Pathological information of LUAD patients for PDX model construction. a HE and immunohistochemical staining (CK7, TTF1, NAPSINA, CK5/6, P63, SYN and P40) on one LUAD patient tissue sections. b The mutation sites in tumor tissue of the LUAD patient in a. c HE staining on the other LUAD patient tissue section. d The information of EGFR mutation in tumor tissue of the LUAD patient in c. [file 12943_2022_1519_MOESM4_ESM.tif]

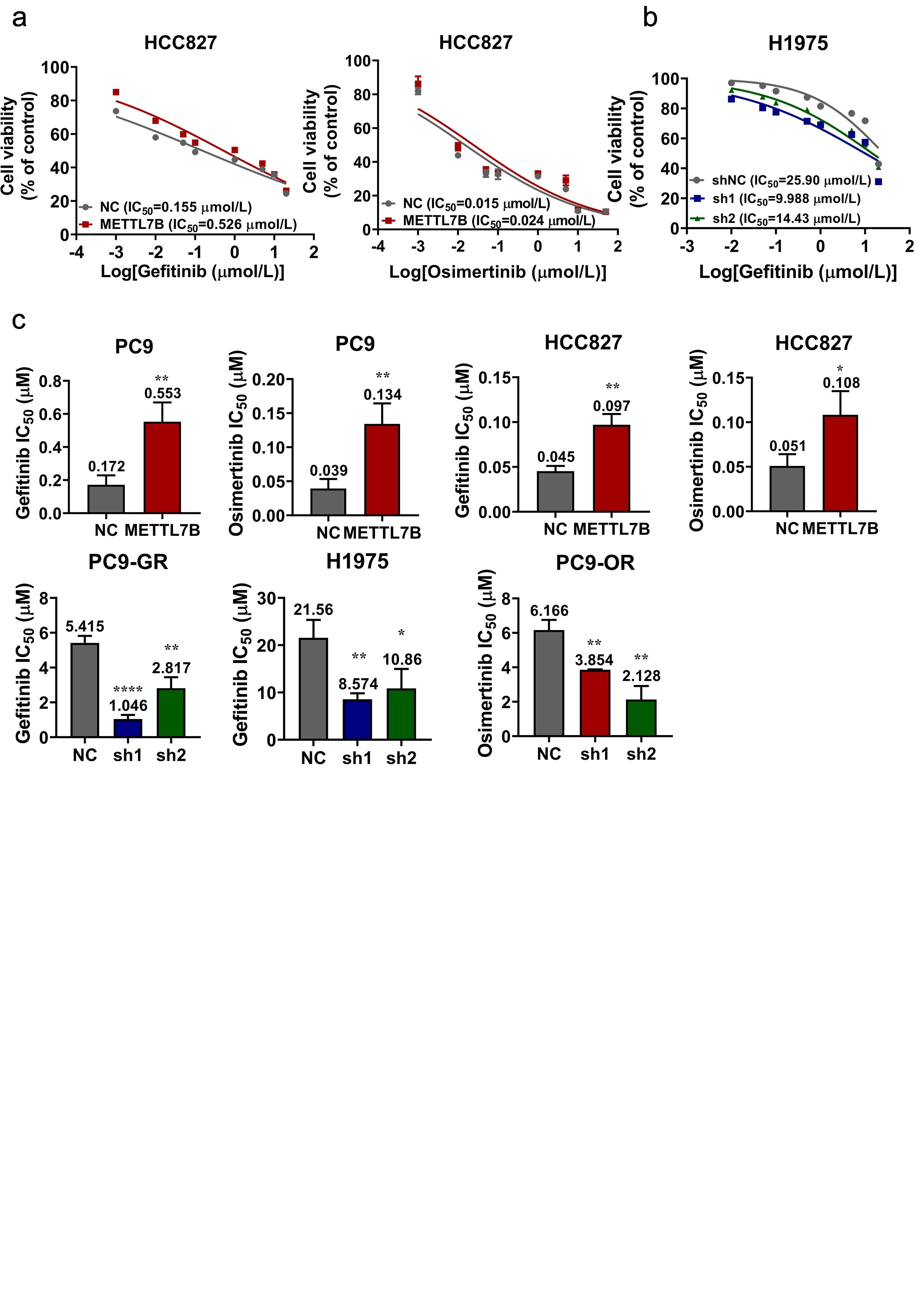

Supplement: Supplementary file 5 — Additional file 5: Figure S2. The IC50 of TKIs in METTL7B-overexpressed HCC827 and METTL7B-suppressed H1975 cells. a FLAG-NC and FLAG-METTL7B was stably transfected into TKIs-sensitive HCC827 cell, and the cell viabilities were evaluated to measure IC50 of TKIs after treatment with different concentrations of gefitinib and osimertinib for 72 h. b METTL7B-shRNAs were stably transfected into gefitinib-resistant H1975 cell, and the cell viability was evaluated to measure IC50 of gefitinib after treatment with different concentrations of gefitinib for 72 h. c The statistical results of IC50 in METTL7B-overexpressed PC9, HCC827 and METTL7B-suppressed PC9-GR, H1975 and PC9-OR cells. *P < 0.05, **P < 0.01 and ****P < 0.0001. [file 12943_2022_1519_MOESM5_ESM.tif]

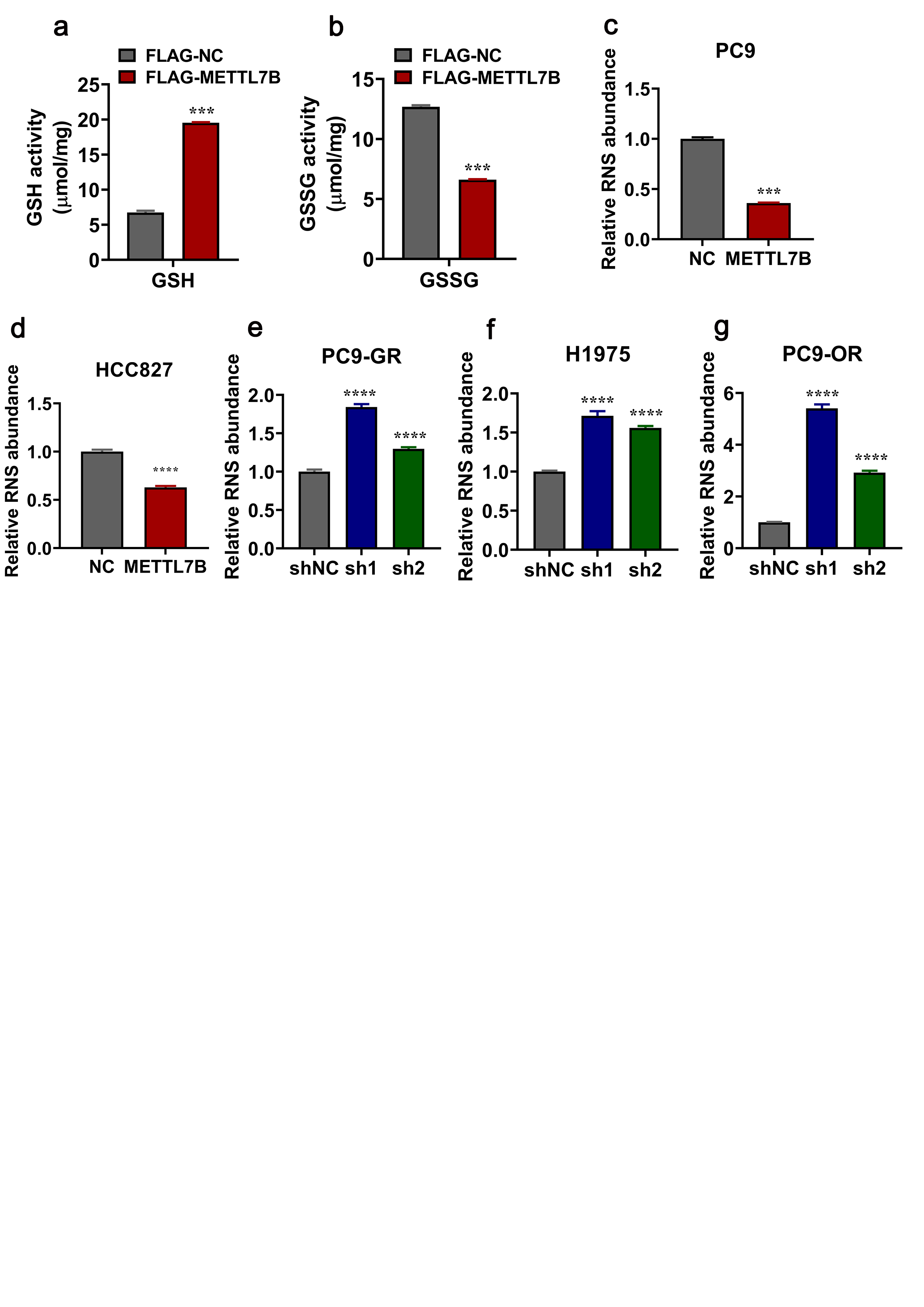

Supplement: Supplementary file 6 — Additional file 6: Figure S3. METTL7B promoted glutathione metabolism in LUAD cells. a and b GSH and GSSG levels were detected in PC9 cells stably transfected with FLAG-NC or FLAG-METTL7B. c-g RNS level was detected in METTL7B-overexpressed PC9 (c), HCC827 (d) and METTL7B-suppressed PC9-GR (e), H1975 (f) and PC9-OR (g) cells. ***P < 0.001 and ****P < 0.0001. [file 12943_2022_1519_MOESM6_ESM.tif]

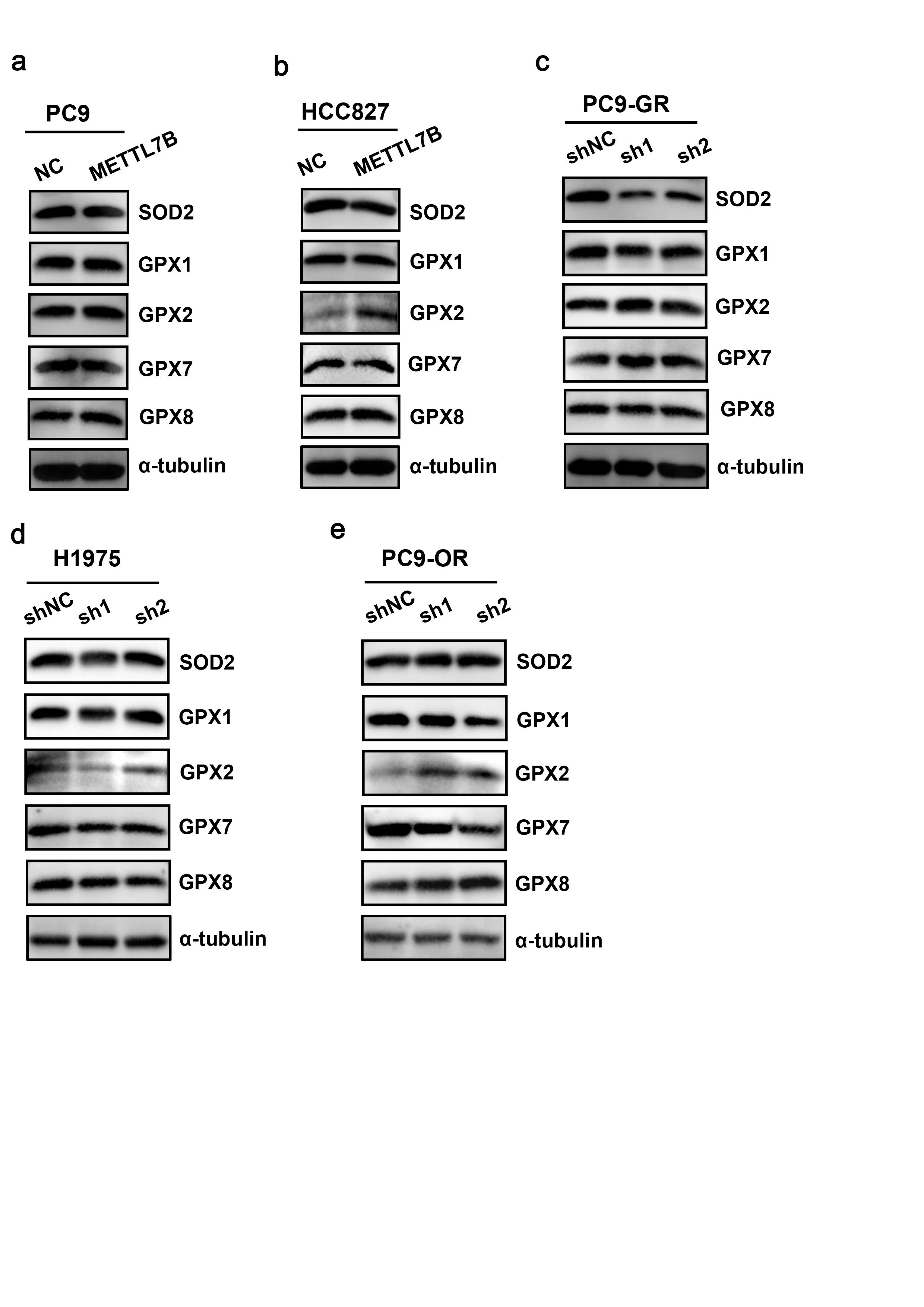

Supplement: Supplementary file 7 — Additional file 7: Figure S4. METTL7B didn’t changed expressions of other antioxidant enzymes. PC9 (a) and HCC827 (b) cells were stably transfected with FLAG-NC or FLAG-METTL7B and the protein levels of SOD2, GPX1, GPX2, GPX7 and GPX8 were measured by Western blot. c-e The same experiment of (a-b) but in METTL7B-suppressed PC9-GR (c), H1975 (d) and PC9-OR (e) cells. [file 12943_2022_1519_MOESM7_ESM.tif]

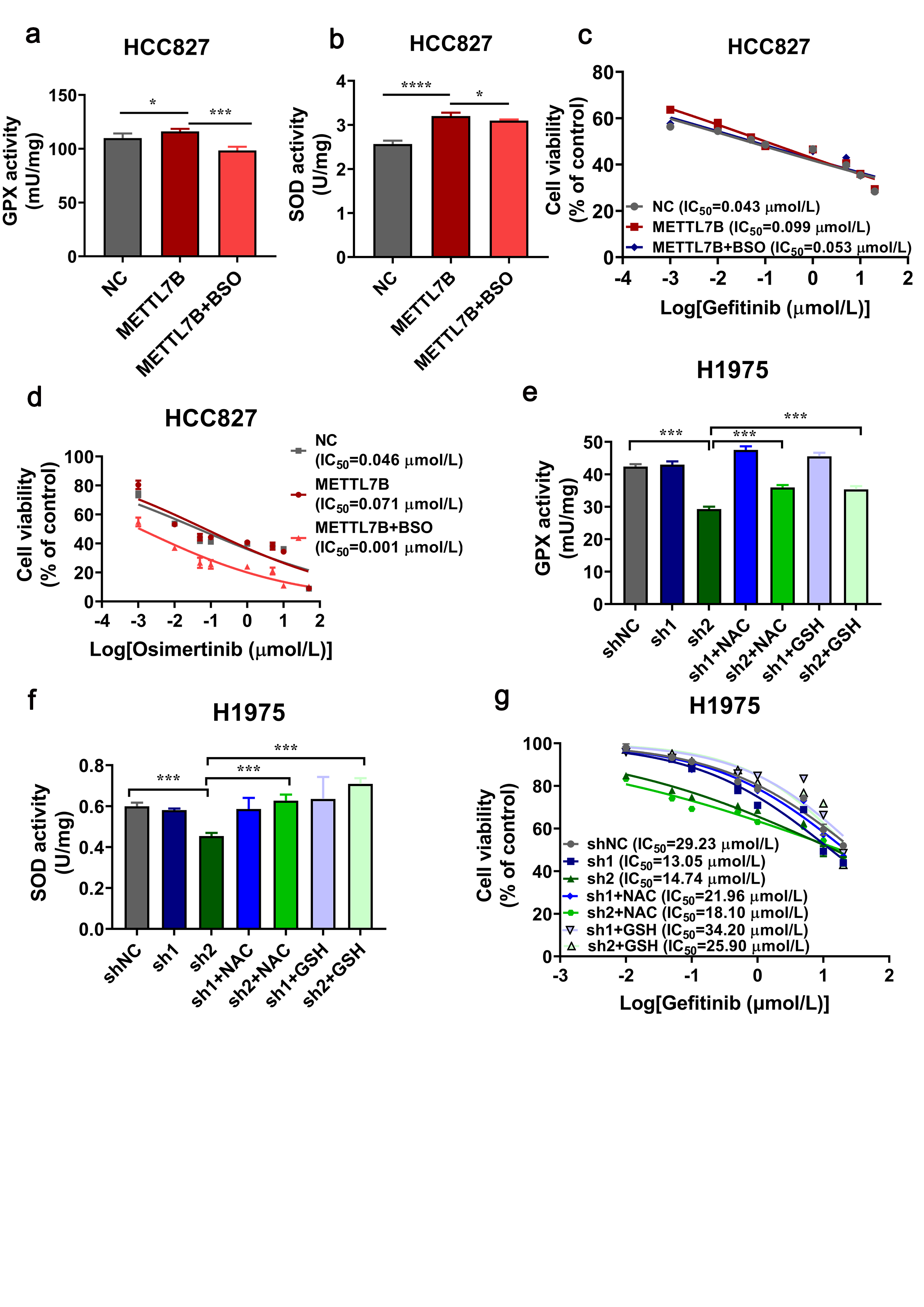

Supplement: Supplementary file 8 — Additional file 8: Figure S5. METTL7B-induced gefitinib resistance was associated with ROS scavenging in LUAD cells. The same experiment of Fig. 6 but in METTL7B-overexpressed HCC827 (a-d) and METTL7B-suppressed H1975 cells (e-g). *P < 0.05, ***P < 0.001 and ****P < 0.0001. [file 12943_2022_1519_MOESM8_ESM.tif]

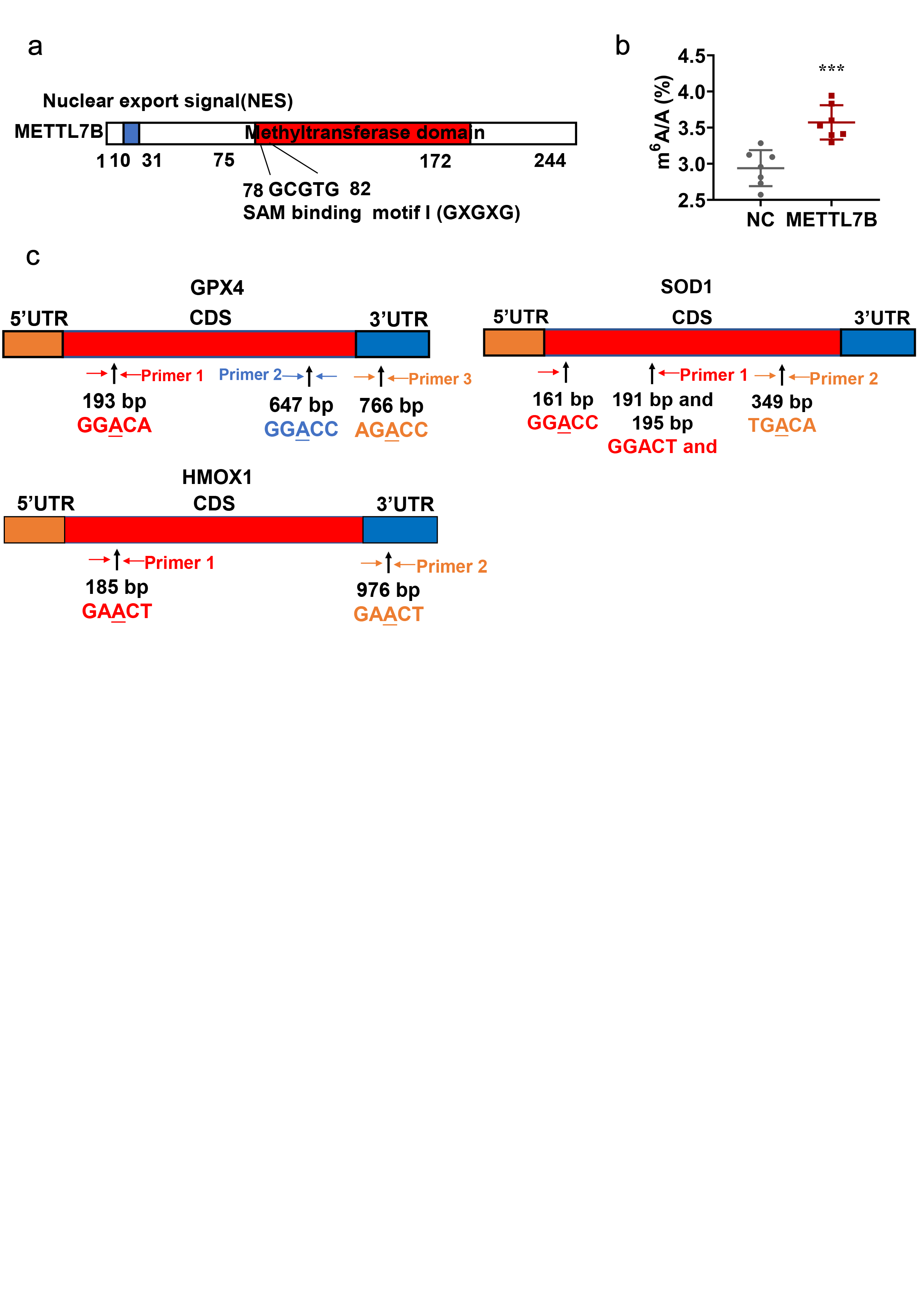

Supplement: Supplementary file 9 — Additional file 9: Figure S6. METTL7B regulated the stability of ROS-scavenging related genes mediated by m6A modification. a The protein domains of METTL7B. b LC-MS/MS quantification of the m6A/A in mRNA of NC and METTL7B-overexpressed PC9 cell. c Sequence analysis of m6A consensus sequence in GPX4, HMOX1 and SOD1 from SRAMP website. The primers for MeRIP-qPCR were presented in Additional file 1: Table S1. ***P < 0.001. [file 12943_2022_1519_MOESM9_ESM.tif]

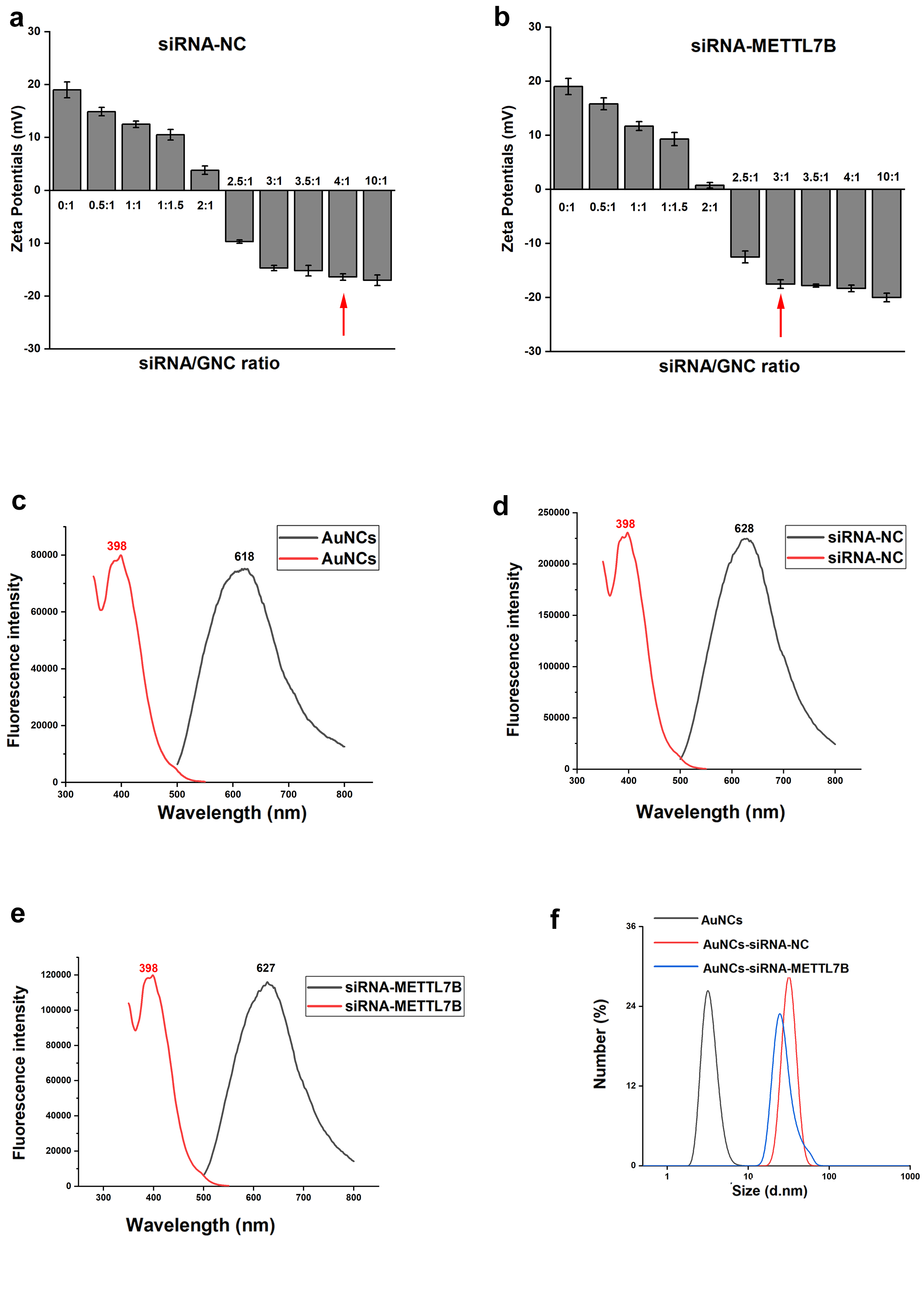

Supplement: Supplementary file 10 — Additional file 10: Figure S7. The characterizations of GNC-siRNA. a and b Characteristics of GNC-siRNA-NC and siRNA-METTL7B complex. c-e Excitation and emission spectrum of GNCs, GNC-siRNA-NC and GNC-siRNA-METTL7B. f Diameter of the prepared GNCs and GNC-siRNA complex. [file 12943_2022_1519_MOESM10_ESM.tif]

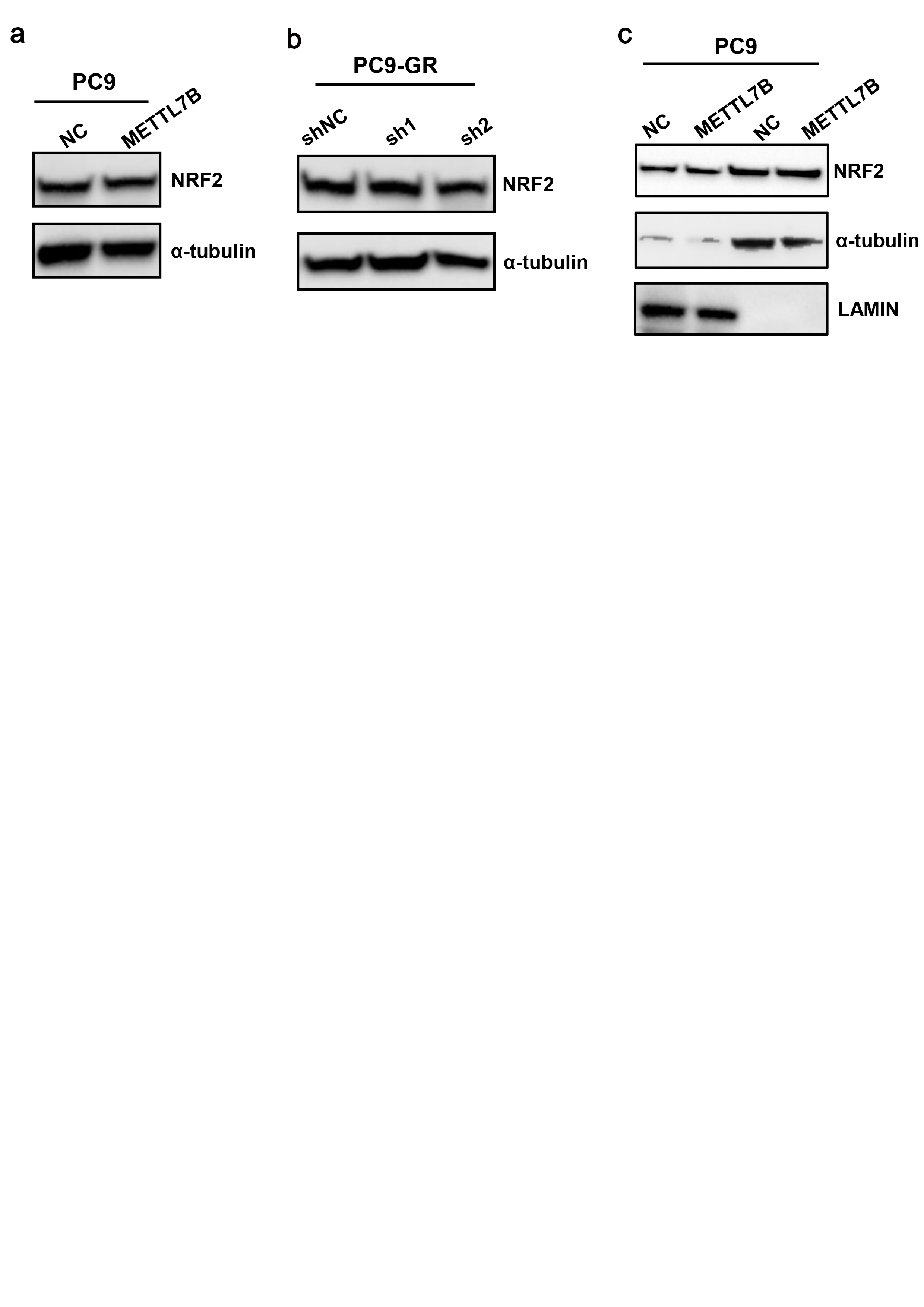

Supplement: Supplementary file 11 — Additional file 11: Figure S8. The expression of NRF2 was not changed by overexpression of METTL7B. a and b FLAG-NC, FLAG-METTL7B (a) or shNC, shMETTL7Bs (b) were stably transfected into PC9 cells or PC9-GR cells, and NRF2 protein levels were measured by Western blot. b Nucleocytoplasmic distribution of METTL7B in PC9 cells stably transfected with FLAG-NC or FLAG-METTL7B. [file 12943_2022_1519_MOESM11_ESM.tif]
